# Translation Inhibition by Rocaglates Activates a Species-Specific Cell Death Program in the Emerging Fungal Pathogen Candida auris

**DOI:** 10.1128/mBio.03329-19

**Published:** 2020-03-10

**Authors:** Kali R. Iyer, Luke Whitesell, John A. Porco, Thomas Henkel, Lauren E. Brown, Nicole Robbins, Leah E. Cowen

**Affiliations:** a Department of Molecular Genetics, University of Toronto, Toronto, Ontario, Canada; b Department of Chemistry and Center for Molecular Discovery (BU-CMD), Boston University, Boston, Massachusetts, USA; c IMAX Discovery GmbH, Dortmund, Germany; University of British Columbia

**Keywords:** *Candida auris*, antifungal, apoptosis, autophagy, cell death, rocaglate, translation

## Abstract

Fungal infections are a major contributor to infectious disease-related deaths worldwide. Recently, global emergence of the fungal pathogen Candida auris has caused considerable concern because most C. auris isolates are resistant to fluconazole, the most commonly administered antifungal, and some isolates are resistant to drugs from all three major antifungal classes. To identify novel agents with bioactivity against C. auris, we screened 2,454 compounds from a diversity-oriented synthesis collection. Of the five hits identified, most shared a common rocaglate core structure and displayed fungicidal activity against C. auris. These rocaglate hits inhibited translation in C. auris but not in its pathogenic relative Candida albicans. Species specificity was contingent on variation at a single amino acid residue in Tif1, a fungal member of the eukaryotic initiation factor 4A (eIF4A) family of translation initiation factors known to be targeted by rocaglates. Rocaglate-mediated inhibition of translation in C. auris activated a cell death program characterized by loss of mitochondrial membrane potential, increased caspase-like activity, and disrupted vacuolar homeostasis. In a rocaglate-sensitized C. albicans mutant engineered to express translation initiation factor 1 (Tif1) with the variant amino acid that we had identified in C. auris, translation was inhibited but no programmed cell death phenotypes were observed. This surprising finding suggests divergence between these related fungal pathogens in their pathways of cellular responses to translation inhibition. From a therapeutic perspective, the chemical biology that we have uncovered reveals species-specific vulnerability in C. auris and identifies a promising target for development of new, mechanistically distinct antifungals in the battle against this emerging pathogen.

## Abstract (precis)

Emergence of the fungal pathogen Candida auris has ignited intrigue and alarm within the medical community and the public at large. This pathogen is unusually resistant to antifungals, threatening to overwhelm current management options. By screening a library of structurally diverse molecules, we found that C. auris is surprisingly sensitive to translation inhibition by a class of compounds known as rocaglates (also known as flavaglines). Despite the high level of conservation across fungi in their protein synthesis machinery, these compounds inhibited translation initiation and activated a cell death program in C. auris but not in its relative Candida albicans. Our findings highlight a surprising divergence across the cell death programs operating in *Candida* species and underscore the need to understand the specific biology of a pathogen in attempting to develop more-effective treatments against it.

## INTRODUCTION

Over the past decade, Candida auris has emerged as a critical threat to our most vulnerable populations, captivating researchers, health care workers, and the media with its environmental persistence, puzzling evolutionary history, and unprecedented resistance to the three major antifungal drug classes: azoles, echinocandins, and polyenes (1, 2). With the pervasiveness of multidrug resistance, the Centers for Disease Control and Prevention classified C. auris at the most serious threat level (3). Genetic modifications associated with resistance include those that modify the drug target, activate drug efflux, or influence resistance mechanisms that remain enigmatic (2, 4–7). Beyond multidrug resistance, the threat posed by C. auris is exacerbated by the pathogen’s ability to persist on abiotic surfaces. This issue is critical in hospital settings where C. auris has been detected on surfaces despite antiseptic treatment, leading to high transmissibility (8–10). Since the discovery of C. auris in 2009, it has been identified across the globe, clustering into four major clades, which are both evolutionarily and geographically distinguishable (5). While recent research efforts have begun to explore the many questions surrounding C. auris, our understanding of the mechanisms governing drug resistance and virulence of this organism remains in its infancy.

A powerful strategy to identify pathogen vulnerabilities that could be exploited therapeutically involves screening compound libraries to identify novel, bioactive chemical matter. Bioactive molecules can then be used as probes for basic biological characterization of the organism and as a foundation for development of therapeutic strategies. Examples of the compounds that have emerged from such screening endeavors include iKIX1, which abrogates azole resistance in Candida glabrata (11); F901318, which inhibits pyrimidine biosynthesis in Aspergillus fumigatus (12); and the indazole derivative Inz-5, which enhances azole activity against Candida albicans (13). In all cases, analyses of these compounds either defined how drug resistance mediators could be targeted to mitigate resistance or identified fungus-specific targets that could be exploited for antifungal drug development. Further, these and other studies highlighted the principle that compounds which trigger fungal cell death as opposed to exerting fungistatic activity provide a therapeutic advantage because they reduce the likelihood that resistance will emerge (14).

Several distinct types of cell death have been reported in fungi, including necrosis, autophagy, and apoptosis (15, 16). Necrosis occurs in response to acute chemical or mechanical damage to the cell, whereas autophagy and apoptosis involve programmed cellular execution. In some fungal species, autophagy is induced as an essential component of pathogenesis (17, 18) or upon nutrient starvation (18, 19). Indeed, prolonged starvation leads to a form of cell death that is dependent on the expression of specific autophagic proteins and proteases (18, 19). Finally, although the topic of apoptosis in a single-celled organism remains controversial, multiple reports have described apoptosis-like responses in fungal species. For example, deletion of cell cycle genes such as *CDC48* (20), expression of mammalian proapoptotic markers such as Bax (21), or treatment with H_2_O_2_ (22) or amphotericin B (23, 24) was shown to lead to phenotypes characteristic of apoptotic cell death. These include membrane disruption and phosphatidylserine exposure, nucleosomal DNA fragmentation, calcium influx, increased levels of reactive oxygen species (ROS), and mitochondrial depolarization (15, 16). While the genetic program(s) responsible for these phenotypes has yet to be established, these phenotypic observations suggest that particular cellular stresses induce active forms of cell death with characteristics similar to those seen with apoptosis in metazoans.

In this study, we screened a diversity-oriented synthetic library, including a subset of natural-product-inspired compounds, to identify novel probes with antifungal activity against C. auris. Among 2,454 compounds screened, the majority of bioactive hits were natural and synthetic variants of the natural-product chemotypes known as rocaglates, which are well-described inhibitors of translation initiation in mammalian cells and Saccharomyces cerevisiae (25). We established that the rocaglates inhibit translation initiation in C. auris, leading to activation of a cell death program with phenotypic characteristics shared with apoptosis in metazoans. In contrast, the related pathogen C. albicans showed inherent resistance to the rocaglates due to an amino acid variant in the drug-binding domain of translation initiation factor 1 (Tif1), the direct target of the compound class. When the species was sensitized through the use of a single *TIF1* point mutation, the rocaglates inhibited C. albicans translation but did not induce cell death. Furthermore, rocaglate-mediated translation inhibition led to hyperacidification and fragmentation of the vacuolar compartment, mitochondrial depolarization, and increased caspase-like activity in C. auris but not in the rocaglate-sensitized C. albicans strain. Our surprising finding that rocaglate-mediated translation inhibition induces cell death in C. auris but not in C. albicans reveals evolutionary divergence in fungal responses to translation inhibition and an actionable vulnerability in an emerging fungal pathogen that poses an urgent threat to human health.

## RESULTS

### Rocaglates exhibit potent single-agent activity against the emerging pathogen C. auris.

**FIG 1:**
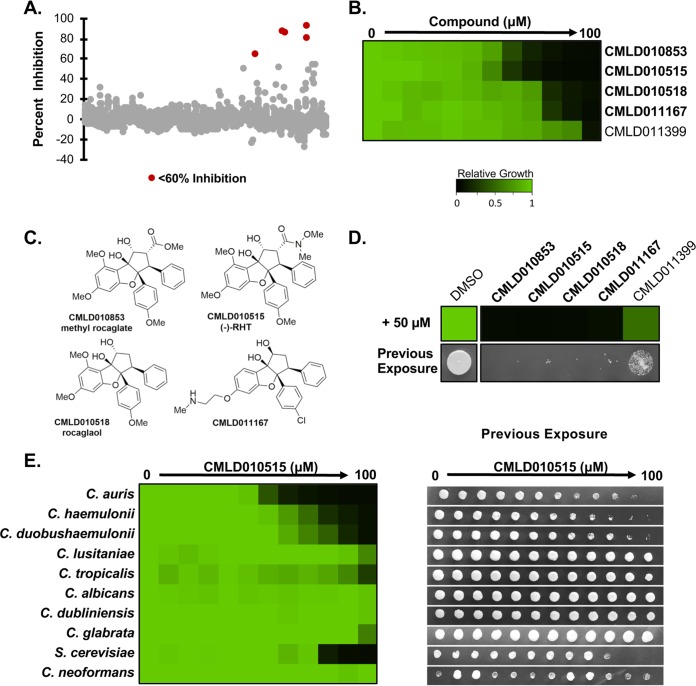
A screen of the BU-CMD library identified rocaglates as having cidal activity against C. auris. (A) The BU-CMD library was screened at 50 μM in RPMI medium at 30°C for 48 h. Five hits (shown in red) were defined as having inhibited growth of C. auris by 60% compared to a DMSO control. (B) The C. auris potency of each hit compound was determined by a dose-response assay in which the titer of each compound was determined at a 2-fold dilution in RPMI medium (*x* axis). Growth was determined by analysis of the optical density at 600 nm (OD_600_) in each well and normalized to that of the DMSO control for each titration (see color bar). Rocaglate compounds are indicated with bold font. (C) Chemical structures of each of the four rocaglates identified from the screen. (D) Growth of C. auris in the absence and presence of the five hit BU-CMD compounds (top panel). Growth was normalized as described for panel B (see color bar). The bottom panel shows the corresponding growth seen when 5 μl of culture from the well was spotted onto YPD agar without drug and incubated for 24 h. (E) Dose-response assays were used to determine the efficacy of one rocaglate compound (CMLD010515) against several fungal pathogens. The titer of the compound was determined at a 2-fold dilution in YPD medium (*x* axis), and the level of growth was measured after 48 h as described above by the use of both normalized OD_600_ assays (left) and spotting assays (right).

To identify novel chemical probes with bioactivity against C. auris, we screened molecules in the Boston University Center for Molecular Discovery (BU-CMD) compound library. This library contained 2,454 molecules obtained through diversity-oriented synthesis techniques as well as semisynthetic natural-product analogs to encompass greater structural diversity and complexity than conventional combinatorial chemistry libraries (26). We screened the BU-CMD library at 50 μM against C. auris, with bioactivity defined by percent growth inhibition for each compound compared to solvent controls. Five hit compounds were identified as inhibiting growth by at least 60% relative to solvent controls (Fig. 1A). The relative potencies of hits from the initial screen were determined by dose-response assays, which confirmed bioactivity for all hit compounds (Fig. 1B). When the chemical structures were assessed, the four most potent hit compounds all shared the cyclopenta[*b*]benzofuran core found in the chemical class consisting of rocaglates (Fig. 1C). The two most potent rocaglates, namely, the natural product CMLD010853 [(-)-methyl rocaglate] (27) and the synthetic rocaglate analogue CMLD010515 [(-)-RHT] (28), are well-studied translation inhibitors, and each displayed antifungal activity against C. auris at concentrations below 12.5 μM. Thus, rocaglates display potent antifungal activity against C. auris.

An alarming characteristic of C. auris is its rapid acquisition of resistance to xenobiotics, particularly to the fungistatic azole fluconazole (5). Compounds with a fungistatic mode of action are often unable to eradicate microbial populations and exert strong selective pressure for the evolution of drug resistance (14). To determine if screening hits had fungistatic or fungicidal activity against C. auris, we used tandem assays consisting of antifungal susceptibility testing followed by spotting onto rich medium without inhibitor. While all of the hit compounds reduced the growth of C. auris after 48 h, only the four rocaglate compounds exhibited cidal activity (Fig. 1D). As the rocaglates displayed such promising activity against C. auris, we next assessed their activity against a range of fungal pathogens, as antifungal activity has been reported previously against plant fungal pathogens (29). Dose-response assays were performed against nine human fungal pathogens ordered by their rough phylogenetic distance from C. auris, followed by spotting assays to evaluate cidality (Fig. 1E). CMLD010515 had reduced bioactivity against fungal pathogens closely related to C. auris, such as Candida haemulonii and C. duobushaemulonii, but no antifungal activity against the majority of more distantly related species. One exception was S. cerevisiae, for which the rocaglate did have fungicidal activity (Fig. 1E), consistent with previous reports for this chemotype (30). These results demonstrate species specificity in rocaglate antifungal activity.

### The rocaglates target the translation initiation factor eIF4A in C. auris.

**FIG 2:**
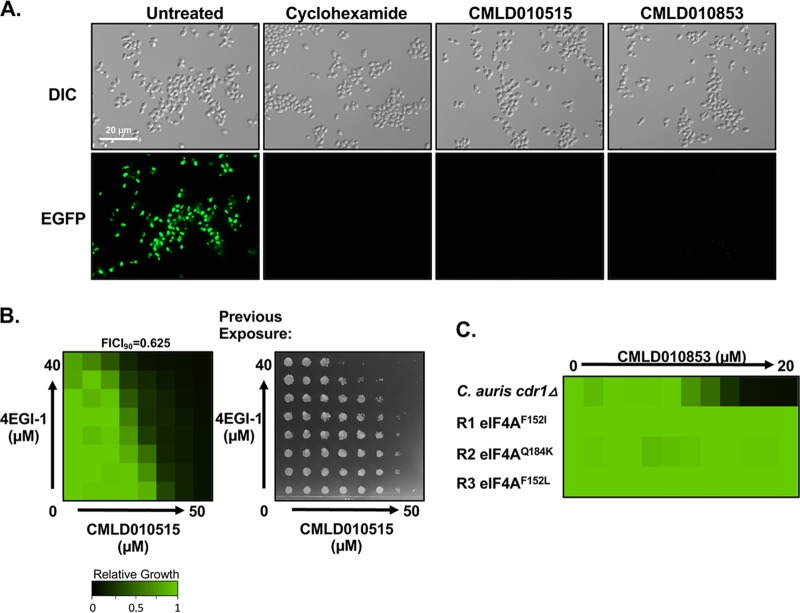
The rocaglates inhibit translation and target the initiation factor eIF4A in C. auris. (A) A Click-iT protein synthesis assay kit was used to visualize protein translation. Cells were subcultured and then treated for 10 min with 10 μg/ml of the translation inhibitor cycloheximide or a 50 μM concentration of each rocaglate identified from the screen, as indicated. The l-homopropargylglycine (HPG) alkyne methionine analog was added, and then the cells were fixed. The azide fluorophore was added, and cells were imaged on the GFP channel to detect if translation had occurred. (B) A dose-response matrix was used to show the interaction between two compounds. The titer of an eIF4G inhibitor, 4EG1-1, was determined in a 2-fold dilution (*y* axis), and that of the rocaglate compound CMLD010515 was determined as indicated on the *x* axis. Cells were grown in YPD medium for 48 h, and growth was measured by determination of the optical density at 600 nm. Growth was normalized to that of the no-drug control (see color bar). The fractional inhibitory concentration index at 90% growth inhibition (FICI_90_) was calculated, with values between 0.5 and 1 indicating an additive interaction. Cells were taken from the checkerboard plate, and 0.5 μl was spotted onto YPD agar medium without drug. Plates were incubated at 30°C for 24 h and imaged. (C) A C. auris-sensitized (*cdr1* Δ) strain was used to select for resistant mutants on YPD agar plates containing a 10 μM concentration of the rocaglate compound. The degree of resistance of C. auris mutants was assessed in YPD medium by dose-response analysis performed with CMLD010853. Growth was determined as described for panel B. Sanger sequencing of *eIF4A* was used to confirm mutations in the gene for all resistant isolates shown (the identified amino acid substitutions are indicated for each strain).

Rocaglates have been reported to inhibit translation initiation in mammalian cells and S. cerevisiae through interaction with orthologues of the eukaryotic initiation factor 4A (eIF4A) (30–33). In examining the rocaglates present in the BU-CMD collection, we found that all four hit rocaglates are among the strongest enhancers of human eIF4A1-polypurine RNA clamping (34), further supporting the identification of translation as a putative target. To determine if the rocaglates identified in our screen inhibit translation in C. auris, we used a fluorescence-based translation assay. When translation is unhindered, an alkynylated methionine analog is incorporated into newly translated proteins, yielding readily detected fluorescent signal upon Click reaction with a green fluorescent (GF) azide (35). Control-treated C. auris cells displayed strong green fluorescence, whereas cultures treated with the known translation inhibitor cycloheximide did not (Fig. 2A). Treatment with each of our rocaglate hits blocked translation as evidenced by loss of fluorescent signal (Fig. 2A; see also Fig. S1A in the supplemental material), supporting the conclusion that these rocaglates inhibit translation in C. auris. To further support the idea that the rocaglates inhibit translation, we performed a dose-response matrix (checkerboard) consisting of a gradient of rocaglate in combination with a gradient of another translation initiation inhibitor, 4EGI-1, as combinations of two compounds targeting the same pathway often elicit additive effects (36). Indeed, these compounds displayed an additive interaction in terms of both the fractional inhibitory concentration index at 90% growth inhibition (FICI_90_) (0.625) and cidality (Fig. 2B). Finally, to determine in an unbiased manner whether the rocaglates inhibit translation initiation through binding to eIF4A, we performed experiments with a drug-sensitized C. auris strain to select for resistance mutations. This strain had the efflux pump gene *CDR1* deleted, allowing lower concentrations of compound to be used (Fig. S1B). Resistant mutants were assessed for their degree of rocaglate resistance to CMLD010853 (methyl rocaglate; Fig. 2C) and for cross-resistance to fluconazole by dose-response assays in order to eliminate strains likely harboring mutations conferring enhanced multidrug efflux (Fig. S1C). Sanger sequencing of the *EIF4A* homolog in C. auris identified mutations in *EIF4a* in all independently derived resistant lineages (Fig. 2C). Thus, the rocaglates operate through their canonical mechanism of translation initiation inhibition in C. auris by binding to eIF4A.

10.1128/mBio.03329-19.2 FIG S1 Rocaglates inhibit translation in C. auris. (A) A Click-iT protein synthesis assay kit was used to visualize protein translation upon treatment with rocaglate compounds identified from the BU-CMD screen against C. auris. The assay was performed as described in the Fig. 2 legend with cycloheximide as an established translation inhibitor. (B) Susceptibility of the C. auris *cdr1* null strain (right) compared to the parental strain (left) to four rocaglate compounds was tested using dose-response assays, as described in the Fig. 1 legend. (C) Fluconazole sensitivity of rocaglate-resistant mutants from solid-selection experiments was assessed using dose-response assays, as described in the Fig. 1 legend. The divergent residue identified by Sanger sequencing of the drug target is indicated for each resistant mutant. Download FIG S1, PDF file, 0.5 MB. Copyright © 2020 Iyer et al. 2020 Iyer et al. This content is distributed under the terms of the Creative Commons Attribution 4.0 International license.

### Rocaglate species specificity is governed by variation in the target binding pocket.

**FIG 3:**
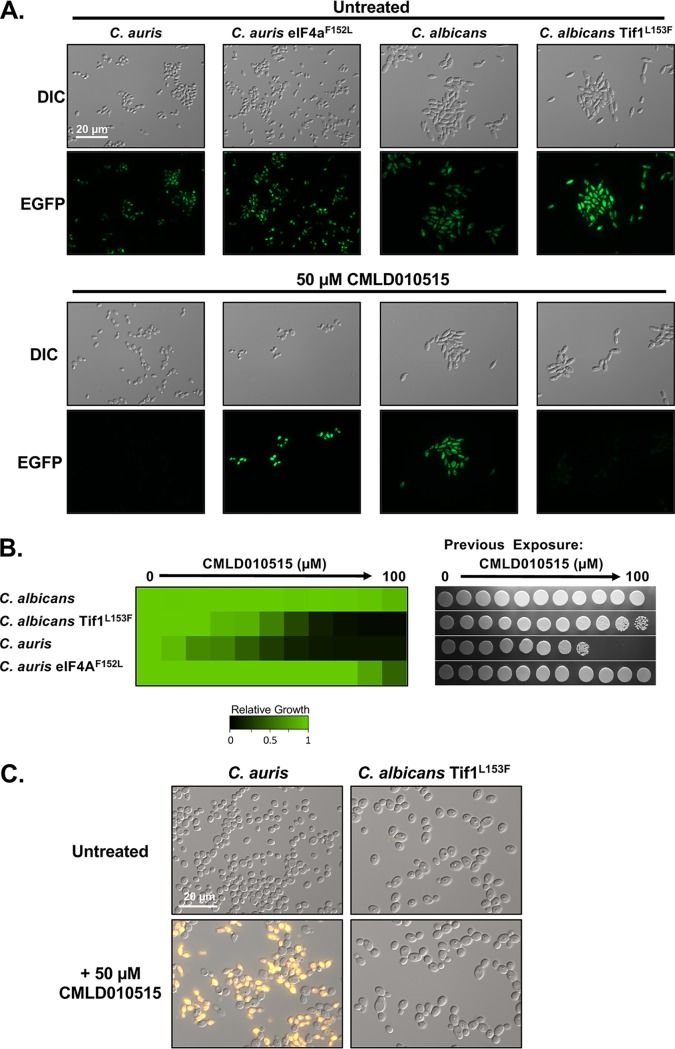
C. albicans is resistant to the rocaglates due to an altered amino acid in Tif1. (A) A Click-iT protein synthesis assay kit was used to visualize protein translation upon rocaglate treatment for rocaglate-sensitive and rocaglate-resistant C. auris and C. albicans strains, as indicated. The parental C. auris strain contains a nourseothricin *N*-acetyl transferase (NAT) marker to match the eIF4A^F152L^ strain. The assay was performed as described for Fig. 2. (B) The degree of resistance of C. auris and C. albicans strains was assessed by dose-response assays, as described for Fig. 1 (left panel). Cells were taken from the MIC assays, and 5 μl was spotted onto YPD medium without drug. Plates were incubated at 30°C for 24 h and imaged (right panel). (C) To visualize cell death, propidium iodide staining was used. C. auris and sensitized C. albicans Tif1^L153F^ were subcultured for 18 h in the absence or presence of 50 μM rocaglate CMLD010515. Cells were pelleted and resuspended in PBS with 1 μg/ml propidium iodide. Cells were imaged by differential interference contrast microscopy and by the use of the DsRed channel on a Zeiss Axio Imager.MI microscope, with the exposure time remaining constant between samples.

During characterization of the rocaglates, we noted a lack of bioactivity against many fungal pathogens, including C. albicans (Fig. 1E), and hypothesized the species-specific activity could be due to alterations in the proximal drug target eIF4A, annotated as translation initiation factor 1 (Tif1) in C. albicans. Clustal omega was used to align the known target in Homo sapiens (37) and S. cerevisiae (30) to the homologous sequences in C. auris, C. albicans, and the other fungal pathogens assessed as described in the Fig. 1E legend (Fig. S2). We focused on the six residues for which mutation has been reported to confer rocaglate resistance in S. cerevisiae (30) and noted that C. albicans and closely related species (C. tropicalis, C. dubliniensis, and C. lusitaniae) contained an alternative residue at position 153 whereas all the sensitive species possessed a phenylalanine at the equivalent position (residue 152 in C. auris; Fig. S2A). Notably, C. glabrata and C. neoformans possessed a phenylalanine at this position (Fig. S2A), despite their intrinsic resistance to the rocaglates (Fig. 1E), suggesting that other factors, including permeability or efflux, may be responsible for the inactivity of this compound class against those species. Given the genomic plasticity of *Candida* species, we examined the sequences of 40 C. albicans isolates and 132 C. auris isolates available on FungiDB. All 40 C. albicans isolates examined encoded a leucine at position 153 whereas all 132 C. auris isolates encoded a phenylalanine at position 152 (Fig. S2B). To determine if this residue was sufficient to confer the resistance phenotype, we generated a C. auris strain expressing eIF4A^F152L^ and a C. albicans strain expressing Tif1^L153F^, each with the residue-swapped version as the sole source of eIF4A, and monitored translation using the fluorescent translation assay described in the Fig. 2 legend. As predicted, the C. auris eIF4A^F152L^ strain was resistant to rocaglate-mediated repression of translation, as indicated by green fluorescence, despite the presence of CMLD010515, while translation in the parental C. auris strain was blocked by the rocaglate (Fig. 3A). In contrast, the C. albicans Tif1^L153F^ strain was sensitive to rocaglate-mediated translation inhibition, whereas the C. albicans wild-type strain was resistant, with persistent translation in the presence of compound (Fig. 3A). These results are supported by previous studies performed with human eIF4a, which demonstrated that substitutions of this single amino acid preclude rocaglate engagement with the protein (32). Thus, the amino acid at residue 153 is responsible for the species specificity of rocaglate activity.

10.1128/mBio.03329-19.3 FIG S2 Alignment of *eIF4A* homologs across different species. (A) Clustal omega was used to align the *eIF4A*/*TIF1* homologous protein sequence for each of the nine fungal pathogens represented in Fig. 1E along with the H. sapiens protein sequence. Of the six identified hot spot residues highlighted, only the residue at position 151 (C. albicans position 153) showed divergence (highlighted in yellow). (B) FungiDB was used to assess genetic variation in *eIF4A*/*TIF1* amongst isolates from the same species. The table shows that all 40 of the C. albicans isolates and all 132 of the C. auris isolates possessed a leucine and a phenylalanine, respectively, at the equivalent residue positions. Download FIG S2, PDF file, 0.6 MB. Copyright © 2020 Iyer et al. 2020 Iyer et al. This content is distributed under the terms of the Creative Commons Attribution 4.0 International license.

To determine whether sensitivity to rocaglates in our residue-swapped strains correlated with a cidal mode of action, dose-response assays were performed, followed by spotting of cells onto drug-free medium (Fig. 3B). Surprisingly, the sensitized C. albicans Tif1^L153F^ strain was not killed by exposure to rocaglate (Fig. 3B). To verify that cell death upon CMLD010515 treatment occurred only in C. auris and not in the sensitized C. albicans Tif1^L153F^ cells, we stained with propidium iodide, which is excluded by live cells. Only the sensitive C. auris strain treated with CMLD010515 displayed propidium iodide positivity (Fig. 3C). Thus, even when translation and growth were inhibited to comparable extents, the rocaglates induced cell death only in C. auris and not in C. albicans.

### Rocaglate treatment disrupts vacuolar homeostasis in C. auris.

**FIG 4:**
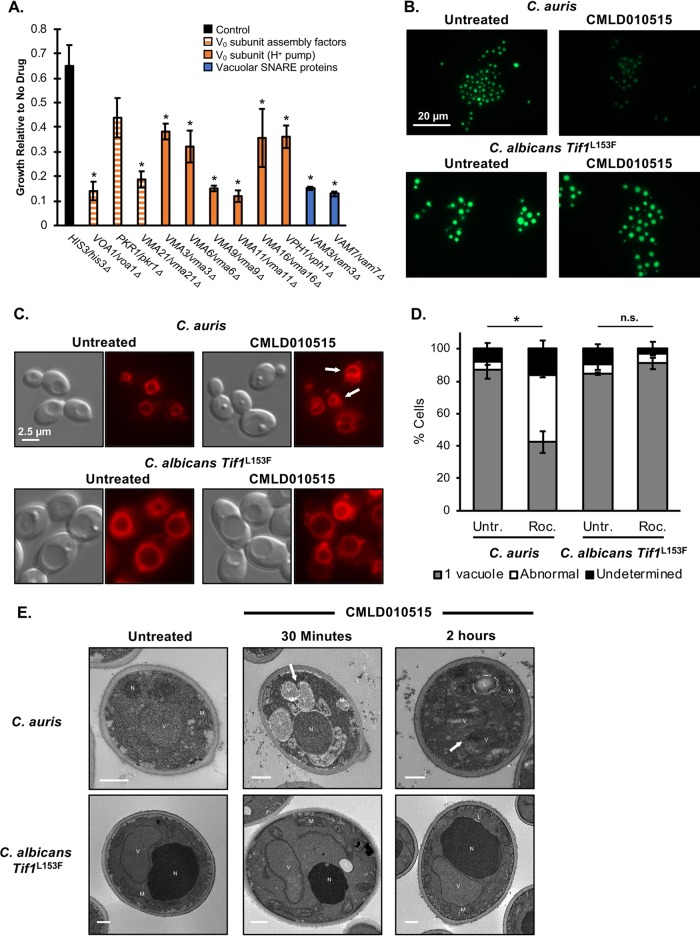
Rocaglate treatment leads to vacuolar hyperacidification and fragmentation in C. auris. (A) S. cerevisiae heterozygous mutants with mutations in genes involved in V-ATPase proton pump function are hypersensitive to rocaglates. Strains were grown in YPD in 96-well plates in the absence and presence of 37.5 μM CMLD010515, and growth was measured at 48 h. Growth of each strain in the presence of the rocaglate was plotted relative to growth in a DMSO control. Error bars indicate standard deviations of results of comparisons between technical triplicates, and a Student's *t* test was used to determine if the relative levels of growth of all strains were significantly different from that seen with the marker matched control (*HIS3*/*his3Δ*) (*P* value of <0.05). (B) Vacuolar pH was monitored using BCECF-AM, an aciditropic dye which becomes trapped in acidic cellular compartments, resulting in an increase in fluorescence intensity that correlates with increased pH. Strains were subcultured in YPD medium buffered to pH 5.5 with MES. Cells were treated with 50 μM CMLD010515 for 60 min and then further incubated with 25 μg/ml BCECF-AM for 30 min. Cells were washed and imaged by differential interference contrast microscopy and by the use of the EGFP channel, with the exposure time remaining constant between samples. (C) Vacuolar morphology was visualized using FM4-64 lipophilic dye. Cells were subcultured for 2.5 h, and then 5 μM FM4-64 was added to the cultures for a 30-min incubation. Cells were washed with YPD, resuspended back to original volume in YPD, and treated with 50 μM CMLD010515 for 90 min. Cells were washed 2× and imaged by differential interference contrast microscopy and by the use of the DsRed channel, with the exposure time remaining constant. Arrows indicate cells with abnormal vacuolar morphology. (D) The number of yeast cells with one vacuole or abnormal vacuolar morphology was quantified over two biological replicates, with ∼100 cells being counted per condition per replicate. Cells with poor staining were classified as “undetermined.” Averages are graphed with the error bars displaying the standard deviations of results of comparisons between replicates. *t* test indicates a significant difference in percentage of cells with abnormal vacuoles in C. auris rocaglate treated samples relative to the untreated control (*, *P* = 0.0046; n.s., not significant). (E) Transmission electron microscopy was used to visualize organelle morphology. Cells were subcultured and then treated with 50 μM CMLD010515. Cells were fixed at time zero (Untretated), 30 min, and 2 h of treatment for imaging. Identifiable organelles are labeled as follows: V, vacuole; N, nucleus; M, mitochondria; L, lipid droplet. Arrows indicate sites of vacuolar fragmentation. Scale bars indicate 500 nM for each respective image.

Next, we sought to characterize the species-selective cell death phenotype that we had observed. To do so, we turned to S. cerevisiae, as this species exhibited the same cidal response to rocaglate exposure as was observed with C. auris (Fig. 1E). We examined a previously reported S. cerevisiae haploinsufficiency (HIP) profile generated with rocaglamide A, a structurally related rocaglate (30). HIP is based on the principle that reduced dosage of a gene encoding a compound’s target or a component of buffering pathways results in hypersensitivity to that compound (38–40). In addition to confirming that strains heterozygous for components of the translation initiation complex were hypersensitive to rocaglate treatment (30), the rocaglamide A HIP profile revealed that the most hypersensitive strain was heterozygous for *VOA1* (30). Voa1 is involved in assembly of the V-ATPase proton pump that maintains an acidic pH within the vacuole (41). By assessing growth of the S. cerevisiae *VOA1*/*voa1Δ* mutant in the presence of CMLD010515, we confirmed that it was hypersensitive to the rocaglate, as were other strains heterozygous for components of the V_0_ sector of the V-ATPase or accessory proteins (*P* value of <0.05; Fig. 4A).

On the basis of these findings, we hypothesized that as a consequence of translation inhibition, the rocaglates alter vacuolar pH, which contributes to their fungicidal activity. To investigate this in C. auris, we used BCECF-AM, an acidotropic dye which accumulates in acidic cellular compartments, exhibiting increased fluorescence intensity in response to increased pH (42, 43). We found that C. auris cells displayed significantly less BCECF-AM staining under conditions of treatment with CMLD010515 than untreated cells; however, the results seen with rocaglate-sensitive C. albicans Tif1^L153F^ did not show as great a loss in intensity upon treatment (Fig. 4B; see also Fig. S3A and B). Additionally, we did not observe reduced fluorescence in the rocaglate-resistant C. auris eIF4A^F152L^ strain, suggesting that the loss of fluorescence was not due to an off-target effect (Fig. S3A and B).

10.1128/mBio.03329-19.4 FIG S3 Rocaglate-sensitive C. auris shows the largest change in vacuolar pH upon rocaglate treatment. (A) Vacuolar pH was observed by monitoring the relative levels of fluorescence of cells stained with BCECF-AM, as described in the Fig. 4B legend. The DIC micrographs are overlaid with EGFP micrographs for C. auris, C. auris eIF4A^F152L^, and C. albicans Tif1^L153F^ for untreated (top panel) and treated (bottom panel) cells. (B) Fluorescence (FITC) of treated cells was quantified on a CytoFlex flow cytometer. For each treatment, 20,000 events were measured, and the fluorescence of each gaited population is shown as a histogram, comparing untreated cells (green) with treated cells (black). The fold change in median fluorescence is indicated at the top right of each histograms. Download FIG S3, PDF file, 1.3 MB. Copyright © 2020 Iyer et al. 2020 Iyer et al. This content is distributed under the terms of the Creative Commons Attribution 4.0 International license.

As a reduction in vacuolar pH negatively regulates vacuolar fusion (44, 45), we explored the impact of rocaglate treatment on vacuole morphology using the vacuolar membrane dye FM4-64. We observed a significant increase in the number of cells with abnormal vacuolar morphology in rocaglate-treated C. auris (*P* value of <0.005); however, we saw no significant differences with respect to vacuolar morphology upon rocaglate treatment of our sensitized C. albicans Tif1^L153F^ strain (Fig. 4C and D). This abnormal morphology appeared consistent with vacuolar fragmentation, which can occur when fission homeostasis and fusion homeostasis are disrupted by hyperacidification of the vacuolar compartment (44). To confirm this phenotype, we used transmission electron microscopy (TEM) to visualize cellular ultrastructures and observed that rocaglate treatment was associated with vacuolar fragmentation in C. auris but not in C. albicans Tif1^L153F^ (Fig. 4E).

Given that vacuolar acidification frequently occurs during autophagy (46), we investigated potential activation of this cell death pathway by rocaglate. Using an Atg8-GFP (Atg8-green fluorescent protein) strain, we saw no induction of autophagy upon rocaglate treatment, unlike the response induced by the classical inducer of autophagy, rapamycin (Fig. S4A). Further, we observed no change in sensitivity to the rocaglates cidal effects in S. cerevisiae autophagy-defective *atg1Δ* or *atg9Δ* mutants (Fig. S4B). Finally, we saw no additive growth inhibitory interaction between the rocaglates and rapamycin in C. auris (Fig. S4C), an effect we would expect if both compounds activated autophagy. Therefore, although increased vacuolar acidification and fragmentation occur in response to translation inhibition in C. auris, autophagy is not the primary process responsible for rocaglate-induced cell death in this organism.

10.1128/mBio.03329-19.5 FIG S4 Rocaglate-induced autophagy is not the mechanism for which cell death occurs in S. cerevisiae and C. auris. (A) An Atg8-GFP S. cerevisiae strain was subcultured for 2 h and then treated with 500 nM rapamycin or 50 μM CMLD010515 for 6 h. Cells were then imaged. When autophagy is not induced, Atg8, a core component of the autophagosome, is localized to the cytoplasm. When autophagy occurs, Atg8 is transported to the vacuoles or concentrated at phagophore assembly sites, as observed upon rapamycin treatment. (B) To determine if cells unable to undergo autophagy were resistant to rocaglate-induced cell death, strains lacking essential autophagy genes (*atg1Δ* or *atg9Δ* strains) were subjected to tandem dose-response and spotting assays, as described in the Fig. 1D legend. (C) The chemical interaction between rapamycin and the rocaglates was assessed by checkerboard assay against C. auris, as described in the Fig. 2B legend. Download FIG S4, PDF file, 1.2 MB. Copyright © 2020 Iyer et al. 2020 Iyer et al. This content is distributed under the terms of the Creative Commons Attribution 4.0 International license.

### Rocaglate-induced cell death displays phenotypic hallmarks of apoptosis.

**FIG 5:**
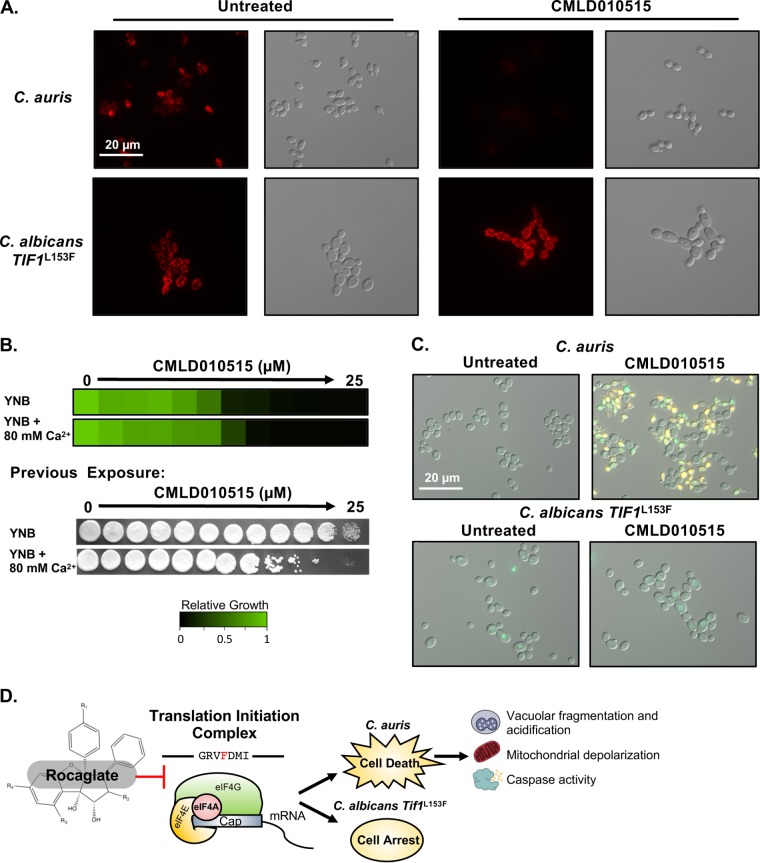
Sensitive C. auris cells display a loss of mitochondrial membrane potential and increased caspase activity. (A) Loss of mitochondrial membrane potential was visualized by the use of MitoTracker Red. Cells were subcultured and then treated with 50 μM CMLD010515 for 3 h at 30°C with agitation. MitoTracker Red was added to the culture at 50 nM, and the cells were further incubated for 1 h. Cells were imaged by differential interference contrast microscopy and the use of the DsRed channel on a Zeiss Axio Imager.MI microscope, with the exposure time remaining constant between samples. (B) The sensitivity of C. auris to the rocaglates in YNB medium containing low calcium levels (0.68 mM) or excess calcium (80 mM) was assessed by dose-response assays, as described for Fig. 1 (top panel). Cells were taken from the MIC assays, and 5 μl was spotted onto YPD medium without drug. Plates were incubated at 30°C for 24 h and imaged (bottom panel). (C) To detect caspase activity, a CaspSCREEN assay kit was used. Cells were subcultured for 18 h in the absence or presence of 50 μM CMLD010515. Cells were resuspended in buffer containing the caspase substrate for 45 min. Propidium iodide was added at 1 μg/ml to visualize dead cells. Cells were imaged on the EGFP channel for caspase activity (green) and on the DsRed channel for death (yellow) and by differential interference contrast microscopy on a Zeiss Axio Imager.MI microscope, all at constant exposure. (D) The rocaglates inhibit translation initiation complex member eIF4A, and the inhibition is contingent on the presence of specific residues in the drug binding pocket. The inhibition leads to cell death in C. auris, in which phenotypes such as vacuolar hyperacidification and fragmentation, mitochondrial depolarization, and increased caspase-like activity are observed. In contrast, rocaglate treatment results in cell arrest in C. albicans in the absence of features of cell death programs.

Phenotypes associated with metazoan apoptosis have been observed in pathogenic fungi under conditions of exposure to various environmental stresses (22–24). To investigate whether these phenotypes occur in rocaglate-treated C. auris, we first monitored loss of mitochondrial membrane potential upon rocaglate treatment using both MitoTracker Red, a dye that is irreversibly trapped within the matrix of mitochondria with intact membrane potential, and the reversible cationic dye tetramethylrhodamine ethyl ester (TMRE), which accumulates in mitochondria due to their negative charge but is released upon depolarization. Untreated C. auris and C. albicans Tif1^L153F^ strains displayed clear staining of their mitochondria upon incubation with either stain (Fig. 5A; see also Fig. S5). Upon rocaglate treatment, however, only the C. auris cells failed to accumulate the dyes in their mitochondria (Fig. 5A; see also Fig. S5), suggesting rocaglate-induced impairment of C. auris mitochondrial function or integrity. No changes in dye staining were observed in C. albicans Tif1^L153F^ or C. auris eIF4A^L152F^ strains treated with CMLD010515. Thus, the rocaglates specifically induced mitochondrial dysfunction via translation inhibition in C. auris, consistent with activation of a species-specific cell death program (Fig. 5A; see also Fig. S5).

10.1128/mBio.03329-19.6 FIG S5 C. auris loses mitochondrial membrane potential over time with rocaglate treatment. Loss of mitochondrial membrane potential was visualized by the loss of staining with the cationic dye TMRE. C. auris, C. albicans Tif1^L153F^, and C. auris eIF4A^F152L^ were subcultured, TMRE was added at a concentration of 100 nM, and cells were incubated for 30 min. Cells were then treated with 50 μM CMLD010515 for up to 4 h at 30°C with agitation. Cells were washed and resuspended in PBS. Cells were imaged by differential interference contrast microscopy and by the use of the TexasRed channel, with the exposure time between samples remaining constant. Download FIG S5, PDF file, 2.7 MB. Copyright © 2020 Iyer et al. 2020 Iyer et al. This content is distributed under the terms of the Creative Commons Attribution 4.0 International license.

Next, we determined whether rocaglate-induced cell death was mediated by calcium signaling, as changes in cytosolic calcium levels have been implicated in triggering cell death in response to chemical stress (47–49). To address this, we performed dose-response assays with the rocaglates against C. auris in medium with minimal levels of calcium (0.68 mM) and with high levels of calcium supplementation (80 mM) that did not affect growth alone. Although we observed no difference in the levels of rocaglate-mediated growth inhibition, spotting to assess cidality revealed a greater-than-4-fold enhancement of rocaglate-mediated cell death in medium supplemented with calcium (Fig. 5B). This result suggests that altered calcium homeostasis modulates C. auris cell death in response to rocaglate treatment.

To further characterize the cell death program, we examined caspase-like enzyme activity and nucleosomal DNA fragmentation. Caspases are classically the major executors of programmed cell death in mammalian cells, and the yeast metacaspase 1 gene (*MCA1*) has been implicated in cell death in S. cerevisiae (22). We engineered a C. auris *mca1* mutant but found no change in the level of rocaglate-mediated cidality (Fig. S6A). Reasoning that an alternative caspase-like activity could be involved, we next took a biochemical approach and looked for increased cysteine-aspartyl protease activity upon treatment of C. auris and of rocaglate-sensitized C. albicans Tif1^L153F^ with CMLD010515 for 18 h. After treatment, the pan-caspase substrate (aspartyl)2-rhodamine 110 (D_2_R) was applied, which, upon proteolytic cleavage within cells, acquires bright green fluorescence. Propidium iodide was also included to simultaneously monitor cell viability. Only C. auris cells treated with the rocaglate CMLD010515 demonstrated an increase in either green or red signal, reflecting activation of proteolytic activity or cell death, respectively (Fig. 5C). In addition to the many dead (red) cells present in the culture, a distinct population of cells was present which exhibited only green fluorescence, suggesting the induction by rocaglate treatment of proteolytic activity (either caspase like or potentially vacuolar in origin), which precedes death in C. auris (50). To complete our survey of apoptotic phenotypes, we assessed whether rocaglates induce nucleosomal DNA fragmentation in C. auris by the use of a terminal deoxynucleotidyltransferase-mediated dUTP-biotin nick end labeling (TUNEL) assay. Intriguingly, we saw no increase in the number of double-stranded breaks in cells treated with the rocaglates (Fig. S6B). Thus, rocaglate treatment induces a unique form of programed cell death in C. auris that leads to phenotypes previously linked to apoptosis in fungi but that is distinct from both traditional apoptosis and autophagy.

10.1128/mBio.03329-19.7 FIG S6 Some phenotypes characteristic of apoptosis are not observed in rocaglate-treated C. auris. (A) To determine if the yeast metacaspase 1 (*MCA1*) homolog in C. auris is required for cell death, a deletion mutant was engineered and subjected to tandem dose-response and spotting assays, as described in the Fig. 1D legend, in comparison with the parental strain. (B) A TUNEL assay was used to observe double-stranded breaks in C. auris cells. Cells were treated with 50 μM CMLD010515 for 4 h and 6 h and fixed with 3.7% (vol/vol) formaldehyde. The cell wall was digested with 24 μg/ml Zymolyase 100T, and then 10 μl of the cell suspension was applied to a microscope slide and allowed to dry. Cells were then incubated in permeabilization solution. The positive control of DNaseI was added to cells on microscope slides. Finally, the slides were incubated with the TUNEL reaction mixture and imaged by the use of differential interference contrast microscopy and the DsRed channel, with the exposure time between samples remaining constant. Download FIG S6, PDF file, 1.1 MB. Copyright © 2020 Iyer et al. 2020 Iyer et al. This content is distributed under the terms of the Creative Commons Attribution 4.0 International license.

## DISCUSSION

Upon screening a chemical library of 2,454 compounds, we identified the rocaglates as a compound class with potent species-selective fungicidal activity against the emerging multidrug-resistant pathogen C. auris (Fig. 1; see also Fig. 5D). We observed that rocaglates inhibit translation in C. auris but not in its close pathogenic relative C. albicans (Fig. 2; see also Fig. 3). The rocaglates have been explored extensively for their anticancer activity which also results from inhibition of translation, specifically through the targeting of translation initiation factor eIF4A (37, 51–54). The eIF4A protein is part of the eIF4F heterotrimeric complex and operates as an RNA helicase that unwinds 5′ untranslated regions (5’UTR) of mRNAs to enable ribosome scanning (55). Rocaglates enhance the affinity of eIF4A for mRNA, circumventing the requirement for ATP and resulting in disassociation from the eIF4F complex, as well as sequestering free eIF4As, which ultimately prevents ribosome scanning (31, 32). All of the rocaglates identified in our screen were among the strongest enhancers of rocaglate-mediated human eIF4A1-polypurine RNA clamping reported in a recent study of BU-CMD rocaglates, including CMLD010515 (RHT) (28) and CMLD010853 (methyl rocaglate) (27). Given the identification of eIF4A as the rocaglate target in C. auris, we sought to determine why other pathogenic fungi were resistant to this compound class. By aligning eIF4A homologs, we identified residue 153 as divergent between 40 C. albicans isolates and drug-sensitive fungal and mammalian species (see Fig. S2 in the supplemental material). The rocaglates bind within a pocket formed by the eIF4A-mRNA interaction. Structurally, the leucine in C. albicans at position 153 compared to the phenylalanine at the corresponding position in C. auris would be expected to reduce the binding pocket size and impair compound binding, leading to the inherent resistance observed in C. albicans (30, 32). This chemostructural finding raises the issue of why C. auris and C. albicans, despite being related, would be divergent at this residue, leading to inherent resistance of C. albicans to this natural product.

Another interesting observation from our study is that, despite genetic sensitization of C. albicans to rocaglate-mediated translation inhibition, sensitization did not lead to rocaglate-induced cell death such as was observed in C. auris (Fig. 3). One of the best-established forms of active cell death in yeast is autophagy, a conserved catabolic process in which the cell digests itself (56). As autophagy can be induced upon starvation, we hypothesized that translation inhibition could mimic starvation conditions and aberrantly induce autophagy in C. auris, as has been reported in mammalian cells (57). Upon induction of autophagy, changes in vacuolar morphology occur as cellular contents are transported to this organelle for degradation (56).To test our autophagy hypothesis, we investigated vacuolar characteristics and discovered that the vacuolar compartment became hyperacidified in rocaglate-treated C. auris but not in the rocaglate-sensitized C. albicans Tif1^L153F^ strain (Fig. 4B; see also Fig. S3A and B). Hyperacidification could reflect autophagy-associated vacuolar activation, with attendant increases in acidic hydrolase/protease activities, or it could represent a secondary consequence of the deregulation of other pathways caused by rocaglate-mediated impairment of protein synthesis. Whatever the mechanism responsible, we did find the hyperacidified vacuoles in rocaglate-treated C. auris to be fragmented (Fig. 4C and D), consistent with previous work establishing that vacuolar acidity negatively regulates vacuolar fusion (44). However, we observed that deletion of genes required for autophagy in S. cerevisiae had no impact on the cidal response to rocaglate treatment (Fig. S4B). Thus, while autophagy-like phenotypes are observed upon rocaglate treatment, the cell death process triggered by rocaglates does not require the classical autophagy machinery.

In addition to autophagy, phenotypes considered the hallmarks of metazoan apoptosis have also been reported in fungi. Although the genetic circuitry controlling execution of programmed cell responses in fungi remains poorly understood, treatment with H_2_O_2_ (22) or amphotericin B (23, 24) was previously shown to induce cell death, with apoptotic features that included nucleosomal DNA fragmentation, caspase activation, production of reactive oxygen species (ROS), and mitochondrial depolarization (15, 16). Consistent with an apoptosis-like cell death program, we observed loss of mitochondrial membrane potential and increased aspartyl-protease activity in rocaglate-treated C. auris but not in the sensitized C. albicans Tif1^L153F^ strain (Fig. 5A and C). Furthermore, we found that the presence of excess extracellular calcium enhanced the cidality of rocaglates against C. auris (Fig. 5B). This calcium sensitivity is consistent with previous reports from studies in which increased calcium influx to the cytosol promoted cell death, specifically by induction of mitochondrial dysfunction (58). As an atypical feature, however, we did not detect DNA fragmentation in C. auris cells treated with rocaglates as monitored by TUNEL assay (Fig. S6B), suggesting either that rocaglates do not induce this classical hallmark of apoptosis in this pathogen or that the specific assay conditions were not optimal for detecting it.

Overall, our findings indicate that rocaglate-mediated translation inhibition induces a noncanonical form of programmed cell death in C. auris that has attributes of both autophagy and apoptosis. A key issue that remains is why translation inhibition triggers these phenotypes. Autophagic features could be a consequence of misrecognition by C. auris signaling pathways of stalled translation as a state of amino acid limitation or pseudostarvation. Phenotypes associated with apoptosis could be a consequence of translation inhibition causing rapid depletion of one or more short-lived antiapoptotic proteins which then allows initiation of an active cell death program. Consistent with this model, the induction of programmed cell death by rocaglates in cancer cells (57, 59, 60) is thought to be driven at least in part by a dramatic reduction in the levels of short-lived prosurvival proteins such as MCL-1, MDM2, and BCL-xL (59, 61). In certain malignancies, this proapoptotic effect is amplified by the fact that unlike translation elongation inhibitors, rocaglates selectively reduce the production of proteins that require eIF4A for synthesis due to their long, highly structured 5′ UTR’s. In mammalian cells, highly eIF4A-dependent proteins include MYC, MDM2, and cyclins, all of which promote malignant transformation. Thus, in cancers, rocaglates not only trigger apoptosis but also impair translation of oncogenic drivers (61). In the context of C. auris, it remains to be explored whether rocaglate-induced cell death is also due to depletion of specific proteins or if it is attributable more broadly to activation of starvation responses as a consequence of impaired protein synthesis.

An urgent need to identify vulnerabilities in C. auris exists and is driven by its extreme resistance to xenobiotic stress. This natural resistance to chemical challenge could be due to the fact that, unlike other *Candida* species that are gut colonizers, C. auris is associated with the skin, where it would be exposed to a plethora of environmental stressors (62). Alternatively, there may be environmental reservoirs of C. auris where frequent exposure to xenobiotics has selected for the emergence of stress tolerance and multidrug resistance (63). Here, we report that targeting translation initiation may provide a strategy to combat this emerging pathogen. The rocaglates have shown activity against various xenograft tumors in mice (59) and in mouse models of malaria (64), indicating that the scaffold’s whole-animal pharmacology is compatible with systemic treatment applications. To fully exploit rocaglates for the treatment of fungal infections, however, development of a more extensively fungus-selective analogue to avoid host toxicity in the context of acute fungal infections will likely be required. Despite the inherent challenges with respect to achieving species selectivity, the rocaglate scaffold is highly tractable to analogue synthesis and precedents exist for the development of fungus-selective molecules targeting highly conserved cellular regulators as a powerful approach to combat drug-resistant fungal infections (65, 66). Given that we found in this study that resistance to the rocaglates was readily acquired (Fig. 2C), a thorough examination of *in vivo* efficacy and of the rate at which resistance emerges in a clinical context should be prioritized with future fungus-selective molecules. Given the limited arsenal of antifungals currently available and the rapid emergence and spread of drug resistance, it is crucial to pursue development not only of broad-spectrum antifungals but also of effective strategies for treatment of infections caused by specific emerging fungal threats.

## MATERIALS AND METHODS

### Culture conditions.

All strains were archived in 25% glycerol in yeast extract-peptone-dextrose (YPD) medium (1% yeast extract, 2% peptone, and 2% d-glucose) and maintained at −80°C. Strains were grown either in YPD medium alone or in YPD medium buffered with MES (morpholineethanesulfonic acid; BioShop), in synthetic defined (SD) medium (0.17% yeast nitrogen base without ammonium sulfate, 0.1% glutamic acid, 2% d-glucose, supplemented with additional histidine-HCl at 20 mg/liter), or in RPMI medium (10.4 g/liter RPMI 1640, 3.5% MOPS [morpholinepropanesulfonic acid], 2% d-glucose, supplemented with additional 5 mg/ml histidine as required, pH 7). All strains, plasmids, and oligonucleotides used in this study are listed in Tables S1, S2, and S3 in the supplemental material.

10.1128/mBio.03329-19.8 TABLE S1 Strains used in this study. Download Table S1, PDF file, 0.1 MB. Copyright © 2020 Iyer et al. 2020 Iyer et al. This content is distributed under the terms of the Creative Commons Attribution 4.0 International license.

10.1128/mBio.03329-19.9 TABLE S2 Plasmids used in this study. Download Table S2, PDF file, 0.06 MB. Copyright © 2020 Iyer et al. 2020 Iyer et al. This content is distributed under the terms of the Creative Commons Attribution 4.0 International license.

10.1128/mBio.03329-19.10 TABLE S3 Primers used in this study. Download Table S3, PDF file, 0.06 MB. Copyright © 2020 Iyer et al. 2020 Iyer et al. This content is distributed under the terms of the Creative Commons Attribution 4.0 International license.

### BU-CMD library screening conditions.

A total of 2,454 diverse compounds from the Boston University Center for Molecular Discovery (BU-CMD) library were used to identify compounds with antifungal activity against C. auris. All compounds were dissolved in dimethyl sulfoxide (DMSO) at 5 mM. RPMI medium was inoculated with ∼1 × 10^3^ cells/ml of C. auris (CaLC3438) from a saturated culture. Medium was dispensed at 100 μl per well into 96-well plates. A 1- μl volume of DMSO-solubilized compound from the library was added into each well to reach a final concentration of 50 μM. Cells were incubated for 48 h at 30°C, and the optical density at 600 nm (OD_600_) was read (Molecular Devices SpectraMax Plus 384). Percent growth was calculated relative to that seen with the DMSO controls on each plate. After the initial screen, all secondary chemical susceptibility assays were performed on fresh sample aliquots that were first assessed for purity by ultraperformance liquid chromatography–mass spectrometry–evaporative light-scattering detector (UPLC-MS-ELSD) analysis.

### Chemical susceptibility assays.

Compound potency was assessed by dose-response assays in 96-well, flat-bottom microtiter plates (Sarstedt) or in 384-well, flat-bottom microtiter plates (Corning) as previously described (67). Plates were incubated at 30°C for the indicated time period. Growth was quantified by measuring OD_600_, and the results were corrected for background media. All strains were assessed in biological-duplicate experiments with technical duplicates. Growth was normalized to the levels seen with the untreated controls, and data were plotted as a heat map using Java TreeView 1.1.6r4.

Dose-response matrixes (checkerboard assays) were performed in a similar manner except that the titers of 2-fold serial dilutions of compound 1 (indicated along the *x* axis of the checkerboard) and compound 2 (indicated along the *y* axis) were determined. Values representing the fractional inhibitory concentration index at 90% growth inhibition (FICI_90_) were calculated as previously described (68).

BU-CMD hit compounds were newly supplied and dissolved in DMSO, 4EGI-1 (Tocris Biosciences) and rapamycin (BioShop) were dissolved in DMSO, fluconazole (Sequoia Research Products) was dissolved in sterile double-distilled water (ddH_2_O), and calcium chloride (BioShop) was used to supplement yeast nitrogen base (YNB) media, as indicated.

### Cidality assays.

Spotting onto drug-free medium was performed to evaluate cidality as previously described (69). Plates were incubated at 30°C and photographed after 24 h. Propidium iodide staining was used to visualize cell death. Cells were subcultured from a saturated overnight culture to an OD_600_ of 0.1 in YPD medium and grown for 18 h in the absence or presence of 50 μM CMLD010515. Cells were pelleted and resuspended in phosphate-buffered saline (PBS) with 1 μg/ml propidium iodide. Cells were imaged by differential interference contrast (DIC) microscopy and by the use of the DsRed channel on a Zeiss Axio Imager.MI microscope (Carl Zeiss) at the same exposure time.

### Fluorescent translation assay.

To observe translation in fungal cells, a Click-iT protein synthesis assay kit (ThermoFisher) was used per the manufacturer’s instructions. Details are provided in Text S1 in the supplemental material. Cells were imaged by DIC microscopy and by the use of the enhanced green fluorescent protein (EGFP) channel on a Zeiss Axio Imager.MI microscope (Carl Zeiss) at the same exposure time.

10.1128/mBio.03329-19.1 TEXT S1 Supplemental methods and supplemental references. Download Text S1, PDF file, 0.2 MB. Copyright © 2020 Iyer et al. 2020 Iyer et al. This content is distributed under the terms of the Creative Commons Attribution 4.0 International license.

### Selection experiments.

To identify mutations conferring resistance to rocaglates, a sensitized C. auris strain (*cdr1*△) was used. Four independent lineages were generated. A total of 1 × 10^8^ cells, quantified by hemocytometer, from each lineage were plated on YPD agar plates containing 10 μM CMLD010853. Plates were incubated at 30°C until colonies were observed (3 to 5 days). Sanger sequencing of *eIF4A* using oligonucleotides oLC7201 to oLC7203 was used to identify mutations.

### BCECF-AM and FM4-64 staining and quantification.

To observe the pH of yeast vacuoles under conditions of rocaglate treatment, the cell-permeative aciditrophic dye BCECF-AM (Sigma) was used as previously described (43). To visualize the vacuolar morphology, the membrane dye FM4-64 was used. Details are provided in Text S1. Cells were imaged by DIC microscopy or with the DsRed channel or with the EGFP channel on a Zeiss Axio Imager.MI microscope (Carl Zeiss), with the exposure time remaining constant between samples of the same species. Brightness was quantified on a CytoFlex flow cytometer (Beckman) when indicated. The number of yeast cells in each sample with one vacuole or with abnormal vacuolar morphology was quantified over two biological replicates. Cells with poor staining or that did not appear within the field of view were classified as undetermined.

### Transmission electron microscopy.

Transmission electron microscopy was used to visualize organelle morphology. Cells were subcultured to an OD_600_ of 0.6 in YPD medium at 30°C with agitation and were then treated with 50 μM CMLD010515. The fixation procedure is described in Text S1. Cells were embedded in the mold at 60°C for 48 h. Cells were sectioned at 90-nm intervals and mounted on a 200-mesh copper grid. Cells were stained with lead citrate for 5 min and then observed with a Hitachi Talos L120C transmission electron microscope at 80 kV.

### Mitochondrial membrane potential assay.

Loss of mitochondrial membrane potential over time was visualized using MitoTracker Red (Molecular Probes) or the reversible cationic dye tetramethylrhodamine ethyl ester (TMRE) (Molecular Probes). Details are provided in Text S1. Cells were imaged by DIC microscopy and by the use of the DsRed (MitoTracker Red) or TexasRed (TMRE) channel on a Zeiss Axio Imager.MI microscope, with the exposure time remaining constant between samples. For the MitoTracker Red-treated cells, brightness was also quantified on a CytoFlex flow cytometer (Beckman).

### TUNEL assay.

DNA fragmentation was visualized using a terminal deoxynucleotidyl transferase mediated dUTP nick end labeling (TUNEL) assay as previously described (70). Details are provided in Text S1. Slides were imaged on a Carl Zeiss microscope on the DsRed channel, and cell fluorescence was quantified by the use of a CytoFlex flow cytometer in the FL2 channel (Ex 561nm/Em 585 nm).

### Caspase-like-activity assay.

To assess caspase-like proteolytic activity, a CaspSCREEN flow cytometric apoptosis detection kit (BioVision) was used per the manufacturer’s instructions. Cells were imaged by DIC microscopy and by the use of the EGFP channel (caspase activity) and the DsRed channel (cell death) on a Zeiss Axio Imager.MI microscope (Carl Zeiss), with the exposure time remaining constant between all samples.

### Flow cytometric analysis for quantification of phenotypes.

To quantify various fluorescent phenotypes, a CytoFlex flow cytometer (Beckman Coulter) was used. In all cases, cells were treated as described for microscopy above. PBS suspensions were diluted 1:10 in PBS, and 250 μl was added to a flat-bottom transparent 96-well plate (Beckman Coulter). Each sample was run using CytExpert software until ∼20,000 events had been documented, populations were gated to exclude debris and doublets, and the median fluorescein isothiocyanate (FITC) value was taken for each sample to determine the median brightness of each cell in that sample.
