# Supplementary figures and images for: Translation Inhibition by Rocaglates Activates a Species-Specific Cell Death Program in the Emerging Fungal Pathogen Candida auris
Source: mBio. 2020 Mar 10;11(2):e03329-19. doi: 10.1128/mBio.03329-19 (PMC7064782; doi:10.1128/mBio.03329-19)

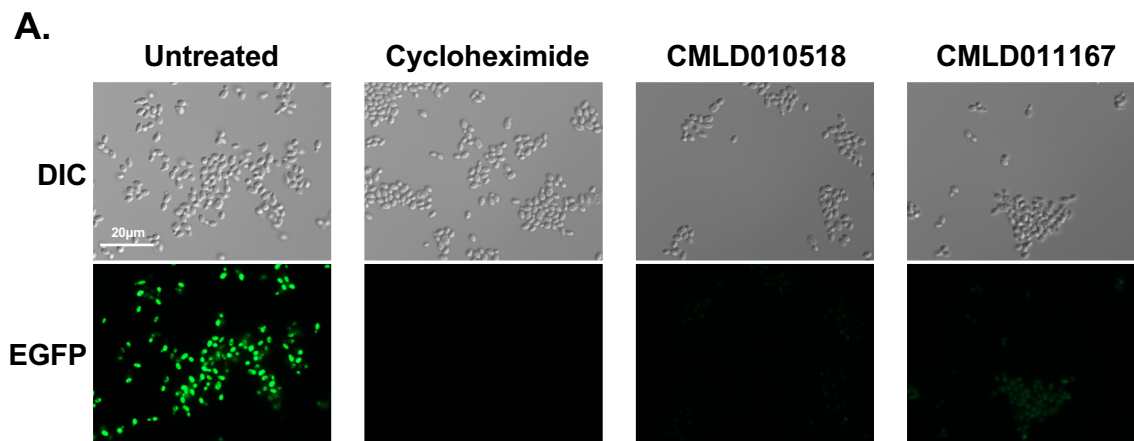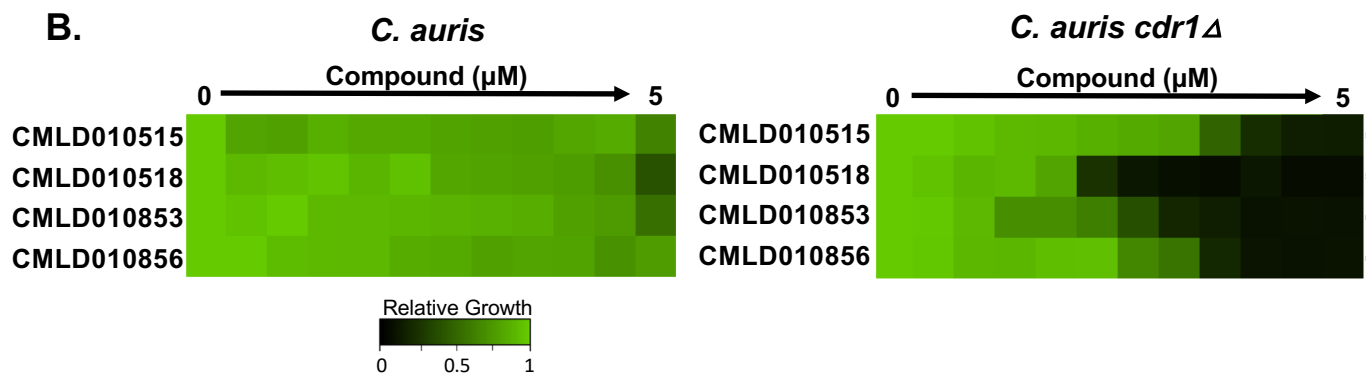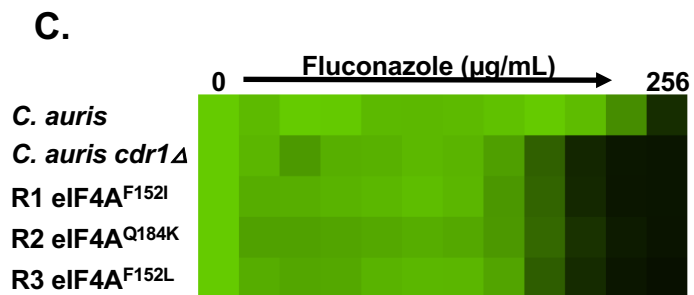

Supplement: FIG S1 [file mBio.03329-19-sf001.pdf]

**A.**

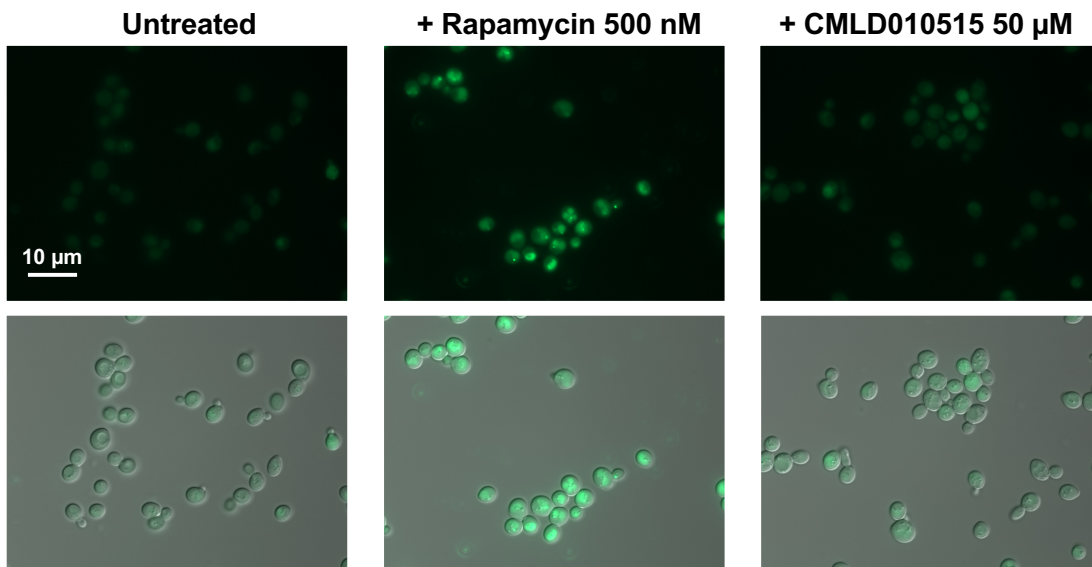

**B.**

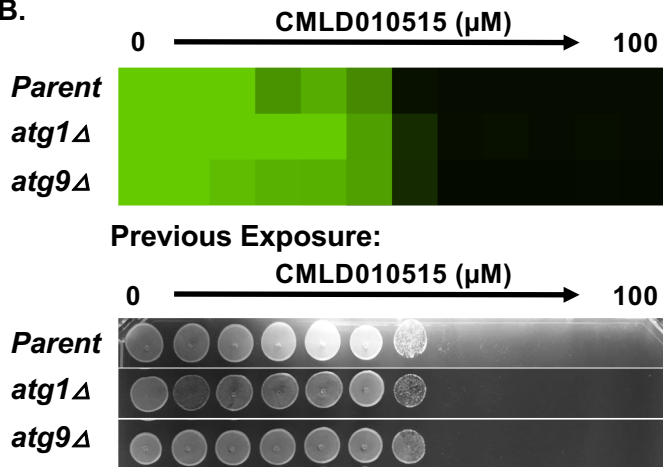

**C.**

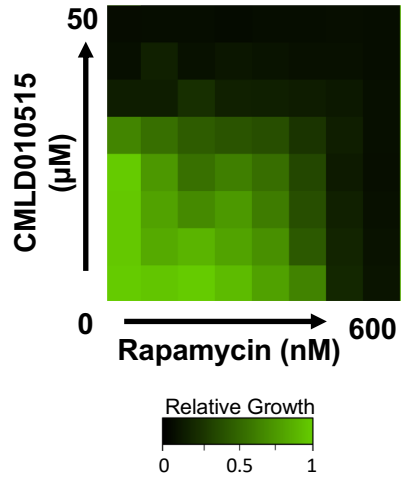

Supplement: FIG S4 [file mBio.03329-19-sf004.pdf]

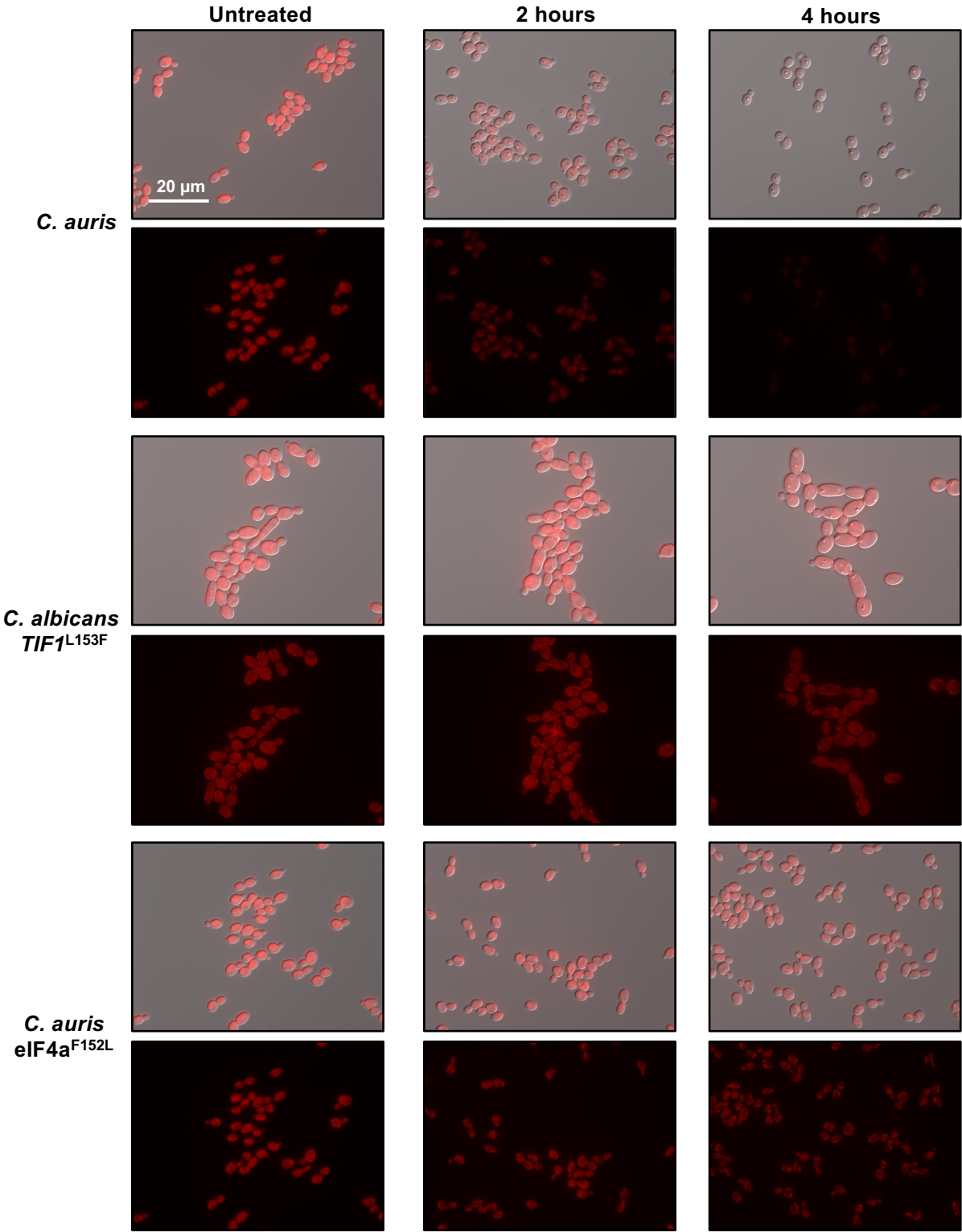

Supplement: FIG S5 [file mBio.03329-19-sf005.pdf]

**A.**

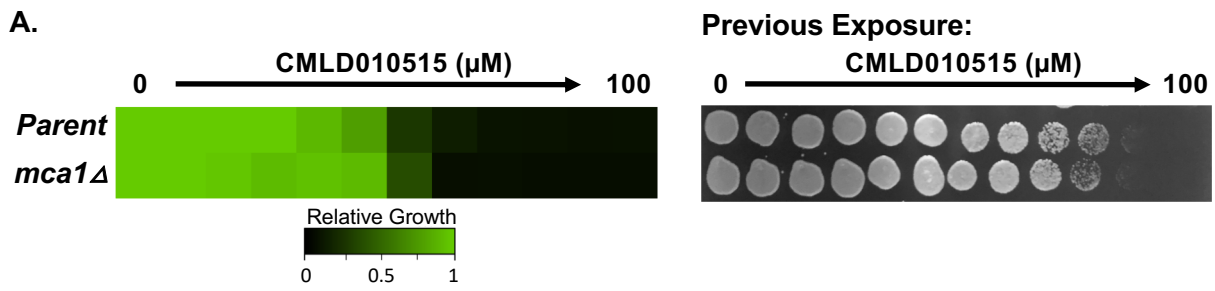

**B.**

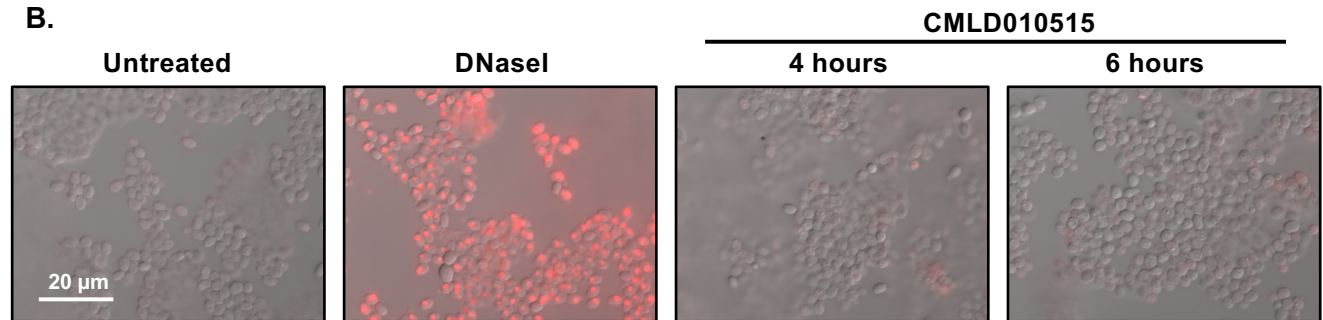

Supplement: FIG S6 [file mBio.03329-19-sf006.pdf]
