# Supplementary material for: Translation Inhibition by Rocaglates Activates a Species-Specific Cell Death Program in the Emerging Fungal Pathogen Candida auris
Source: mBio. 2020 Mar 10;11(2):e03329-19. doi: 10.1128/mBio.03329-19 (PMC7064782; doi:10.1128/mBio.03329-19)
Supplement: FIG S2 [file mBio.03329-19-sf002.pdf]

A.

```
                                                                     146/147 151
C.tropicalis         ITAIGLYLNVTVHASIGGTSMSDDIEAFR-SGVQIVVGTPGRVYDMIERRYFKTDKVKMF    169
C.albicans           ITAIGLYLKVTVHASIGGTSMSDDIEAFR-SGVQIVVGTPGRVLDMIERRYFKTDKVKMF    169
C.dubliniensis       ITAIGLYLKVTVHASIGGTSMSDDIEAFR-SGVQIVVGTPGRVLDMIERRYFKTDKVKMF    169
C.lusitaniae         ISHIGLYLNVTVHASIGGTSMSDDIEAFR-SGVQIVVGTPGRVSDMIERRYFKTHKVKMF    168
C.auris              ISHIGLYLKVSVHASIGGTSMADDIEAFR-SGVQIVVGTPGRVFDMIDRRYFKTHKVKMF    168
C.haemulonii         IKHIGLYLKVSVHASIGGTSMADDIEAFR-SGVQIVVGTPGRVFDMIDRRYFKTHRVKMF    168
C.duobushaemulonii   IKHIGLYLKVSVHASIGGTSMADDIEAFR-SGVQIVVGTPGRVFDMIDRRYFKTHRVKMF    168
C.glabrata           VMALGFHMDIKVHACIGGTSFVEDAEGLR-D-AQIVVGTPGRVFDNIQRRKFKVDNIKMF    168
S.cerevisiae         VMALAFHMDIKVHACIGGTSFVEDAEGLR-D-AQIVVGTPGRVFDNIQRRRFRTDKIKMF    167
C.neoformans         VIALGDYLNVDCHACVGGTAVREDIARLN-EGPHIVVGTPGRVFDMINRGALKTEAVMMF    174
H.sapiens            VMALGDYMGASCHACIGGTNVRAEVQKLQMEAPHIIVGTPGRVFDMLNRRYLSPKYIKMF    179

                                 180 183 187
C.tropicalis         ILDEADEMLSSGFKEQIYNIFRLLPETTQVVLLSATMPQEVLEVTTKFMNNPVRILVKKD    229
C.albicans           ILDEADEMLSSGFKEQIYNIFRLLPETTQIVLLSATMPQDVLEVTTKFMNNPVRILVKKD    229
C.dubliniensis       ILDEADEMLSSGFKEQIYNIFRLLPETTQIVLLSATMPQDVLEVTTKFMNNPVRILVKKD    229
C.lusitaniae         ILDEADEMLSSGFKEQIYNIFKLLPETTQVVLLSATMPQDVLEVTTKFMNNPVRILVKKD    228
C.auris              ILDEADEMLSSGFKEQIYNIFKLLPETTQVVLLSATMPQDVLEVTTKFMNNPVRILVKKD    228
C.haemulonii         ILDEADEMLSSGFKEQIYNIFKLLPETTQVVLLSATMPQDVLEVTTKFMNNPVRILVKKD    228
C.duobushaemulonii   ILDEADEMLSSGFKEQIYNIFKLLPETTQVVLLSATMPQDVLEVTSKFMNNPVRILVKKD    228
C.glabrata           ILDEADEMLSTGFKEQIYQIFTMLPPTTQVVLLSATMPRDVLEVTAKFMQNPVRILVKKD    228
S.cerevisiae         ILDEADEMLSSGFKEQIYQIFTLLPPTTQVVLLSATMPNDVLEVTTKFMRNPVRILVKKD    227
C.neoformans         CLDEADEMLSTGFKESIYEIFQLLPGETQVVLLSATMAPEVLDVTKKFMRDPIRILVKKD    234
H.sapiens            VLDEADEMLSRGFKDQIYDIFQKLNSNTQVVLLSATMPSDVLEVTKKFMRDPIRILVKKE    239
```

B.

Species	# Isolates Assessed	Amino Acid at Residue 151
C. albicans	40	L
C. auris	132	F
