# Supplementary material for: Translation Inhibition by Rocaglates Activates a Species-Specific Cell Death Program in the Emerging Fungal Pathogen Candida auris
Source: mBio. 2020 Mar 10;11(2):e03329-19. doi: 10.1128/mBio.03329-19 (PMC7064782; doi:10.1128/mBio.03329-19)
Supplement: FIG S3 [file mBio.03329-19-sf003.pdf]

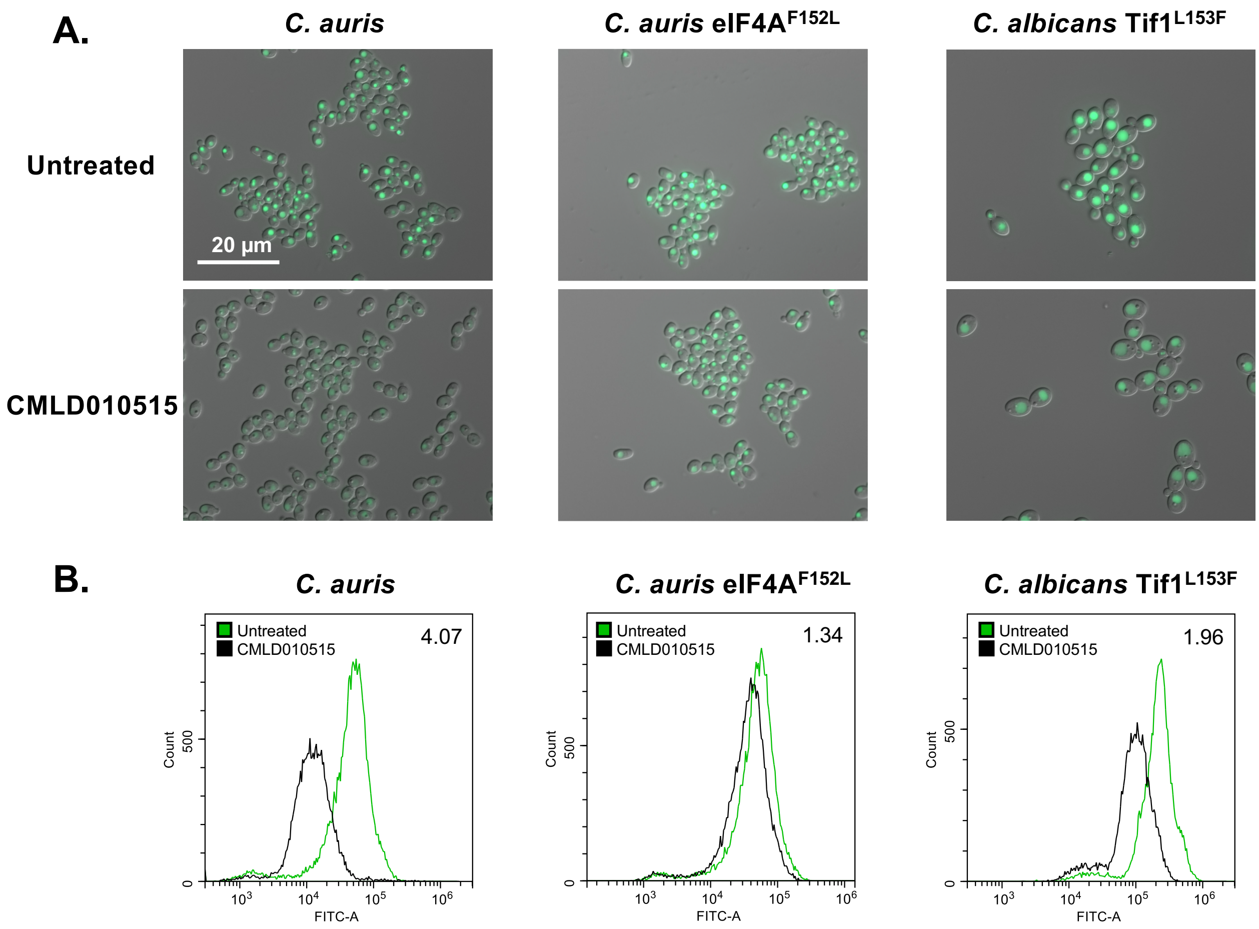
A.
C. auris
C. auris eIF4A^F152L
C. albicans Tif1^L153F
Untreated
CMLD010515
20 µm
B.
C. auris
C. auris eIF4A^F152L
C. albicans Tif1^L153F
Untreated
CMLD010515
4.07
1.34
1.96
Count
FITC-A
