# Supplementary material for: Translation Inhibition by Rocaglates Activates a Species-Specific Cell Death Program in the Emerging Fungal Pathogen Candida auris
Source: mBio. 2020 Mar 10;11(2):e03329-19. doi: 10.1128/mBio.03329-19 (PMC7064782; doi:10.1128/mBio.03329-19)
Supplement: TABLE S1 [file mBio.03329-19-st001.pdf]

Supplementary Table S1: Strains used in this study

Strain ID	Description	Genotype	Source
CaLC3438	*C. auris* VPCI 673/P/12	Clinical isolate	Chowdary Lab
CaLC5083	*C. auris* Ci6684	Clinical isolate	(1)
ChLC3447	*C. haemulonii* M3	Clinical isolate	Chowdary Lab
CdLC3482	*C. duobushaemulonii* M20	Clinical isolate	Chowdary Lab
ClLC572	*C. lusitaniae* ATCC 42720 reference strain	Clinical isolate	(2)
CtLC575	*C. tropicalis* 1685	Clinical isolate	Heitman Lab
CaLC239	*C. albicans* SN95	*arg4Δ/arg4Δ, his1Δ/his1Δ, URA3/ura3Δ::imm434 IRO1/iro1Δ::imm434*	(3)
CdLC268	*C. dubliniensis* MYA-577	Clinical isolate	(4)
CgLC1002	*C. glabrata* BG2	Clinical isolate	(5)
ScLC151	*S. cerevisiae* BY4741	*MATa his3Δ1 leu2Δ0 met15Δ0 ura3Δ0*	(6)
CnLC3142	*Cryptococcus neoformans* H99a	Clinical isolate	(7)
CaLC5280	*C. auris cdr1Δ*	CaLC5083 + *cdr1Δ::NatMX*	(8)
CaLC5921	*C. auris* + CMDL010853 resistant	CaLC5083 + *eIF4A*^{F152I} - *NatMX*	This study
CaLC5922	*C. auris* + CMDL010853 resistant	CaLC5083 + *eIF4A*^{Q184K} - *NatMX*	This study
CaLC5989	*C. auris* + CMDL010853 resistant	CaLC5083 + *eIF4A*^{F152L} - *NatMX*	This study
CaLC5543	*C. albicans* + rocaglate sensitive	*CaLC 239+ Tif1*^{L153F}*/Tif1*^{L153F}	This study
CaLC5813	*C. auris* NAT resistant	CaLC5083 + *eIF4A -NatMX*	This study
CaLC5814	*C. auris* rocaglate resistant	CaLC5083 + *eIF4A*^{F152L} - *NatMX*	This study
Yeast Heterozygous Diploid Library YSC1055	*S. cerevisiae BY4743*	*BY4743 (MATa/α; his3Δ1/his3Δ1; leu2Δ0/leu2 Δ0; met15Δ0/MET15; LYS2/lys2Δ0; ura3Δ0/ura3Δ0) + HIS3/his3Δ::KanMX*	(6)

Yeast Heterozygous Diploid Library YSC1055	*S. cerevisiae BY4743*	*BY4743 + VOA1/voa1Δ::KanMX*	(6)
Yeast Heterozygous Diploid Library YSC1055	*S. cerevisiae BY4743*	*BY4743 + PKR1/pkr1Δ::KanMX*	(6)
Yeast Heterozygous Diploid Library YSC1055	*S. cerevisiae BY4743*	*BY4743 + VMA21/vma21Δ::KanMX*	(6)
Yeast Heterozygous Diploid Library YSC1055	*S. cerevisiae BY4743*	*BY4743 + VMA3/vma3Δ::KanMX*	(6)
Yeast Heterozygous Diploid Library YSC1055	*S. cerevisiae BY4743*	*BY4743 + VMA6/vma6Δ::KanMX*	(6)
Yeast Heterozygous Diploid Library YSC1055	*S. cerevisiae BY4743*	*BY4743 + VMA9/vma9Δ::KanMX*	(6)
Yeast Heterozygous Diploid Library YSC1055	*S. cerevisiae BY4743*	*BY4743 + VMA11/vma11Δ::KanMX*	(6)
Yeast Heterozygous Diploid Library YSC1055	*S. cerevisiae BY4743*	*BY4743 + VMA16/vma16Δ::KanMX*	(6)
Yeast Heterozygous Diploid Library YSC1055	*S. cerevisiae BY4743*	*BY4743 + VPH1/vph1Δ::KanMX*	(6)
Yeast Heterozygous Diploid Library YSC1055	*S. cerevisiae BY4743*	*BY4743 + VAM3/vam3Δ::KanMX*	(6)
Yeast Heterozygous Diploid Library YSC1055	*S. cerevisiae BY4743*	*BY4743 + VAM7/vam7Δ::KanMX*	(6)
ScLC6277	*S. cerevisiae* GFP-*atg8*	*MATα ura3-52 leu2-3,112 his3- 200 trp1- 901 lys2-801*	(9)

		suc2- 9 mel GAL GFP-ATG8::LEU2	
ScLC6320	*S. cerevisiae* Y13206	*MATα snq2Δ::KILeu2; pdr3Δ::KIura3; pdr1Δ::NATMX; can1Δ::STE2pr-Sp_his5 lyp1Δ; his3Δ1 leu2Δ0 ura3Δ0 met15Δ0LYS2+*	(10)
	S. cerevisiae agt1Δ	ScLC6320 *agt1Δ::KANMX*	(10)
	S. cerevisiae agt9Δ	ScLC6320 *agt9Δ::KANMX*	(10)
