# Supplementary material for: Translation Inhibition by Rocaglates Activates a Species-Specific Cell Death Program in the Emerging Fungal Pathogen Candida auris
Source: mBio. 2020 Mar 10;11(2):e03329-19. doi: 10.1128/mBio.03329-19 (PMC7064782; doi:10.1128/mBio.03329-19)
Supplement: TABLE S2 [file mBio.03329-19-st002.pdf]

Supplementary Table S2: Plasmids used in this study

Plasmid ID	Description	Source
pLC963	CaCas9/sgRNA plasmid	(11)
pLC1089	pLC963 + *C. albicans Tif1* sgRNA	This study
pUC19	pUC19	(12)
pLC1049	*NAT* marker	(8)
pLC1105	pUC19 – *eIF4A-NAT*	This study
pLC1106	pUC19 – *eIF4A*^{F153L}*-NAT*	This study
