# Supplementary material for: Translation Inhibition by Rocaglates Activates a Species-Specific Cell Death Program in the Emerging Fungal Pathogen Candida auris
Source: mBio. 2020 Mar 10;11(2):e03329-19. doi: 10.1128/mBio.03329-19 (PMC7064782; doi:10.1128/mBio.03329-19)
Supplement: TABLE S3 [file mBio.03329-19-st003.pdf]

Supplementary Table S3: Oligonucleotides used in this study

Oligo ID	Description	Sequence (5' to 3')
oLC6944	CaTIF1_orf448 -2 F	AAATTGTCGTTGGTACTCCAGGTAGAGTCTTCG ACATGATTGAAAGAAGATATTTCAAAA
oLC6945	CaTIF1_orf448-2 R	TTTTGAAATATCTTCTTTCAATCATGTCGAAGAC TCTACCTGGAGTACCAACGACAATTT
oLC6972	CaTIF1_orf296 F	AACCAGAGAATTGGCTTTGC
oLC6923	CaTIF1_orf663 R	CCAAATTCATGAACAACCCA
oLC7157	CauEIF4a-477 F	TACCTCAAAAGCCCTGTTCC
oLC7158	CauEIF4a+1204 R	GAGTTGCCGAGGTGATCAAT
oLC7153	CauEIF4a-567 F	ACTGTTGCAATGCGTAGTCG
oLC6398	CaSFL1AB+1550-R	TTACTGTCTTCACGTGCACG
oLC274	pJK863down-F	CTGTCAAGGAGGGTATTCTGG
oLC7156	CauEIF4a+1296 R	CGAGAACGGTATTTACTTCG
oLC7157	CauEIF4a-477 F	TACCTCAAAAGCCCTGTTCC
oLC7158	CauEIF4a+1204 R	GAGTTGCCGAGGTGATCAAT
oLC6918	CaTIF1 Repair T	ATTTGATCATGTCTAAGACTCTACCG
oLC6919	CaTIF1 Repair B	AAAACGGTAGAGTCTTAGACATGATC
oLC4609	snr52p-F	GAAACTTCGGCCCAATAGGATTGG
oLC7154	CauEIF4a+212 R	CTATACTGCTGTCGATTCGATACTAACGCCGCC ATCCAGTGCGTCAAGAATAATTGGACG
oLC7155	CauEIF4a+266 F	CGCTGGCCGGGTGACCCGGCGGGGACGAGGCA AGCTTGATTCTGACTCATTCACTAAGTC
oLC6296	pLC605 NAT F	ACTGGATGGCGGCGTTAGTA
oLC6304	pLC605 NAT R	ATCAAGCTTGCCTCGTCC
oLC243	M13-R	CAGGAAACAGCTATGAC
oLC7166	CauEIF4a-197 R	GATGGCTCGAGGTTCAAGAG
oLC244	M13-F	GTAAAACGACGGCCAG
oLC7167	CauEIF4a+823 F	ACCAGGATTGGCAAACAGAC
oLC7200	puc19 MCS F	AATTTCACACAGGAAACAGC
oLC7201	CauEIF4a-88 F	CATACAGTAAGGCGAGTCTC
oLC7202	CauEIF4aorf_465 F	GACAGAAGATACTTCAAGAC
oLC7203	CauEIF4aorf_996 F	GTTATTAACTACGACTTGCC
oLC7204	CauEIF4a+297 F	CGTTTTGACTATTACCCGAG
oLC3854	CaNAT+206-R	GATTCATCATCGGATTCATC
oLC7161	CauEIF4aorf_435 F	GGTACTCCTGGTAGAGTGTTAGACATGATCGAC AGAAGATACTTC
oLC7162	CauEIF4aorf_435 R	GAAGTATCTTCTGTCGATCATGTCTAACACTCT ACCAGGAGTACC
oLC7720	Cauris_MCA1-1032	CTGCTGCGGCTAAGAAGACT
oLC7721	NAT_CaurisMCA1-47	CTATACTGCTGTCGATTCGATACTAACGCCGCC ATCCAGTGAAGCTTGTGCCTGGTTTCT

oLC7722	NAT_CaurisMCA1+2	CGCTGGCCGGGTGACCCGGCGGGGACGAGGCA AGCTTGATGGCCTTAGCAGAGTGTCAGC
oLC7723	Cauris_MCA1+836	GTGTTCCGACAACGGAGTTT
oLC7724	Cauris_MCA1-908	GATTGACTTGTTGGGCTCGT
oLC7725	Cauris_MCA1-908	CCTGCTGAGCAACAACTCTG
oLC7726	Cauris_MCA1-1092	GACCAGGTGCCAAAGTGATT
oLC7727	CaurisMCA1_orf48	AGGTTCCCTTTGGATCGAGT
oLC7728	CaurisMCA1_orf587	CAGCAAGGTTACAACCAGCA
oLC7729	Cauris_MCA1+875	GCATGGCCCTGATGATATTT
