# Supplementary material for: Translation Inhibition by Rocaglates Activates a Species-Specific Cell Death Program in the Emerging Fungal Pathogen Candida auris
Source: mBio. 2020 Mar 10;11(2):e03329-19. doi: 10.1128/mBio.03329-19 (PMC7064782; doi:10.1128/mBio.03329-19)
Supplement: TEXT S1 [file mBio.03329-19-s0001.pdf]

Strain Construction

C. albicans strain CaLC5543 was constructed using the CRISPR Cas9 system previously described.[56] *C. auris* strains were constructed using homologous recombination and an electroporation transformation approach as described previously.[57]

CaLC5543: *C. albicans* Tif1^{L153F}/Tif1^{L153F}

To alter residue 153 from a leucine to a phenylalanine in *C. albicans,* repair primers oLC6944 and oLC6945 containing the nucleotide change from A to C were annealed. pLC1089 was digested with KpnI and SacI and transformed into CaLC239 (SN95) along with the repair piece. Colonies were patched and PCR using oLC6972 and oCL6923 was employed to amplify 400 bp to send for sequencing to confirm base change.

CauLC5813: *C. auris* eIF4A-NAT

The eIF4A-NAT construct was amplified from pLC1105 with oLC7157 and oLC7158. The construct was ethanol precipitated and transformed into *C. auris* CauLC5083 using a standard electroporation protocol and plated on YPD-NAT plates. Integration was verified using primer pairs oLC7153/6398 and oLC274/7156.

CaLC5814: *C. auris* eIF4A^{F152L} NAT

The eIF4A-NAT construct was amplified from pLC1106 with oLC7157 and 7158. The construct was ethanol precipitated and transformed into *C. auris* CauLC5083 using a standard electroporation protocol and plated on YPD-NAT plates. Integration was verified using primer pairs oLC7153/6398 and oLC274/7156.

CaLC6610: *C. auris mca1Δ*:: NAT

To delete *MCA1*, ~1kb upstream and downstream of the gene was amplified using primers oLC7720/oLC7721 and oLC7722/oLC7723 and the NAT marker from pLC1049 was amplified using oLC6296/oLC6304. Fusion PCR using nested primers oLC7724/oLC7725 was used to create the final construct. Integration was verified using primers oLC7720/oLC6308 and oLC274/oLC7723 and absence of wild type band (oLC7727/oLC7728).

pLC1089: To alter residue 153 from a leucine to a phenylalanine in *C. albicans,* pLC963 was digested with BsmBI and ligated with annealed sgRNAs oLC6918 and oLC6919 directed towards *TIF1*. This was transformed into DH5α making pLC1089. Colonies were sequenced to verify guide using oLC4609.

pLC1105: ~1kb homology to the *C. auris* eIF4A locus was amplified: upstream (oLC7153/7154) and downstream (oLC7155/7156) with 40bp overlap to NAT marker. The NAT marker from pLC1049 was amplified using oLC6296/oLC6304. Fusion PCR with nested primers (oLC7157/7158) containing cut sites XmaI for upstream and Sac1 for downstream produced a full length 3.5kb construct. pUC19 and the construct were digested independently with Xma1 and Sac1 overnight and ligated at a ratio of 3:1 intert:vector. 5uL of mixture was transformed into DH5alpha and plated on LB plates with 100 μg/mL ampicillin. Colonies were verified for integration upstream with oLC243/7166 and downstream with oLC244/7167. Correctly genotyped colonies were sequence verified with tiling primers oLC7200-7204 and oLC3854.

pLC1106: From pLC1105, site-directed mutagenesis was performed using oLC7161/7162 as previously described. Dpn1 was used to remove remaining template DNA and 10 ng was transformed into Max Efficiency DH5α. Sanger sequencing was used to confirm the mutation with oLC7163 and then tiling primers were used to confirm no other mutations had occurred (oLC7200-7204 and oLC3854).

Fluorescent Translation Assay

Briefly, cells were grown in minimal YNB medium until log phase (~OD_{600} 0.4) was reached. Cells were then treated for 10 minutes with either 10 μg/mL of the known translation inhibitor cycloheximide (AG Scientific Inc.) or 50 μM of each rocaglate identified from the screen as indicated. The HPG alkyne methionine analog was added to the cells for 30 minutes and then cells were fixed using v/v 70% ethanol for 1 hour rocking. Cells were pelleted by centrifugation and washed twice with 3% BSA in PBS. The reaction cocktail containing the azide fluorophore was used to resuspend pelleted cells at a volume based on the volume of the initial sample. Samples were incubated for 30 minutes in the dark at room temperature. Cells were then washed with the rinse buffer and resuspended in PBS. Cells were imaged by differential interference contrast microscopy and the EGFP channel on a Zeiss Axio Imager.MI (Carl Zeiss) at the same exposure time.

BCECF-AM Staining and Quantification

Briefly, strains were sub-cultured at OD_{600} of 0.2 from a saturated overnight culture at 30°C with agitation for 2.5 hours in YPD medium buffered to pH 5.5 in 2-(N-morpholino)ethanesulfonic acid (MES; Bioshop). Cells were then treated with 50 μM CMD010515 for 60 minutes at 30 °C with

agitation. The cultures were then further incubated under the same conditions with 25 μg/mL BCECF-AM for 30 minutes. Cells were washed 2X in PBS and resuspended in PBS. Cells were either imaged by differential interference contrast microscopy and the EGFP channel on a Zeiss Axio Imager.MI (Carl Zeiss) with the exposure time remaining constant between samples of the same species, or brightness was quantified on a CytoFlex Flow Cytometer (Beckman).

Mitochondrial Membrane Potential Assay

Cells were sub-cultured at OD_{600} of 0.2 from a saturated overnight culture at 30°C with agitation for 2 hours in YPD medium. For TMRE treatment, TMRE was added to cultures at a concentration of 100 nM and cells were incubated for 30 minutes. Cells were then treated with 50 μM CMD010515 for 4 hours at 30°C with agitation. For MitoTracker Red, after the initial sub-culture cells were treated with 50 μM CMD010515 for 3 hours at 30°C with agitation. MitoTracker Red was added to the culture at 50 nM and the cells were further incubated for 40 minutes. For both treatments cells were washed and resuspended in PBS. Cells were imaged by differential interference contrast microscopy and the DsRed (MitoTracker Red) or TexasRed (TMRE) channel on a Zeiss Axio Imager.MI with the exposure time remaining constant between samples. For the MitoTracker Red treated cells, brightness was also quantified on a CytoFlex Flow Cytometer (Beckman).

TUNEL Assay

Yeast cells were sub-cultured at OD_{600} of 0.2 from a saturated overnight culture at 30°C with agitation for 2 hours in YPD medium. Cells were then treated with 50 μM CMD010515 for 4 and 6 hours at 30°C with agitation. At each time point cells were pelleted and fixed with 3.7% (vol/vol)

formaldehyde for 1 hour at room temperature and then washed three times with PBS. The cell wall was digested with 24 μg/mL Zymolyase 100T (10^5 units/g; MP Biomedicals, Irvine, CA) at 37°C for 60 minutes. For microscope visualization, 10 μL of the cell suspension was applied to a microscope slide and allowed to dry for 30 minutes at 37°C. The remaining treated cells were kept within an eppendorf tube. Cells were rinsed with PBS and then incubated in permeabilization solution: [0.1% (vol/vol) Triton X-100 and 0.1% (wt/wt) sodium citrate] for 2 minutes on ice. Cells were rinsed twice with PBS. The positive control of 30 U DNaseI was added to the cells on microscope slides. The slides were placed in a humidified box for 1 hour at 37°C. They were then washed for 15 minutes twice in PBS. Slides were incubated with 10 μL of TUNEL reaction mixture (terminal deoxynucleotidyl transferase and fluorescein isothiocyanate dUTP) for 60 minutes at 37°C. Cells were rinsed three times with PBS and 5 μLof 50% glycerol was added to cells on the slide.

Caspase-like Activity Assay

Cells were sub-cultured to an OD_{600} of 0.1 and grown for 18 hours in the absence or presence of 50 μM rocaglate CMD010515. Cells were pelleted and resuspended in buffer containing the fluorescent caspase substrate. Cells were incubated for 45 minutes, washed, and resuspended in PBS. Propidium iodide was added at 1 μg/mL to visualize dead cells.

Transmission Electron Microscopy

Cells were fixed prior to rocaglate treatment and after 30 minutes or 2 hours of treatment by combining cell culture 1:1 with 2X of a prefixative solution (0.2 M PIPES pH 6.8, 0.2 M sorbitol, 2 mM $MgCl_2$, 2 mM $CaCl_2$, 4% glutaraldehyde in ddH_2O). Cells were incubated in fixative for 5

minutes, spun down at 1500*g* for 5 minutes and then resuspended in 1X prefixative solution. Cells were incubated overnight at 4°C. Cells were then washed 3X in ddH_2O and resuspended in in 5 mL of 2% aqueous solution of potassium permanganate for 5 minutes. Cells were pelleted and resuspended in fresh 2% potassium permanganate and incubated for 45 minutes. Cells were washed with ddH_2O and the En-Bock stain was added by overlaying the cell pellet with 1% uranyl acetate and incubating at room temperature for 1 hour followed by washing 3X with ddH_2O. Celled were dehydrated by pelleting and washing 2X in 30% ethanol for 30 minutes each. This was repeated with 50, 70, and 90% ethanol and finally 3X with 100% ethanol for two hours each. One part Spurr's resin mixed with two parts 100% ethanol was added to the cells for 3 hours using an agitator, followed by two parts Spurr's resin mixed with one part 100% ethanol for 4 hours. 100% Spurr's resin was added overnight using an agitator and finally one more change with fresh Spurr's with agitation for 2 hours.

FM4-64 Staining and Quantification

Rocaglate-sensitive *C. auris* (CaLC5083) and *C. albicans* (CaLC5543) were sub-cultured to an OD_{600} of 0.2 from a saturated overnight culture in YPD medium at 30°C with agitation for 2.5 hours. FM4-64 (ThermoFisher) was added to the cultures to a concentration of 5 µM and shaking incubation was continued for 30 minutes. Cells were washed with YPD, resuspended back to the original volume in YPD, and treated with 50 µM CMLD010515 for a 90 minute outgrowth at 30 °C with agitation. Cells were washed 2X in PBS and resuspended in PBS.

Supplementary References

1. **Chatterjee S**, **Alampalli SV**, **Nageshan RK**, **Chettiar ST**, **Joshi S**, **Tatu US**. 2015. Draft genome of a commonly misdiagnosed multidrug resistant pathogen *Candida auris*. BMC Genomics **16**:686.

2. **Butler G**, **Rasmussen MD**, **Lin MF**, **Santos MAS**, **Sakthikumar S**, **Munro CA**, **Rheinbay E**, **Grabherr M**, **Forche A**, **Reedy JL**, **Agrafioti I**, **Arnaud MB**, **Bates S**, **Brown AJP**, **Brunke S**, **Costanzo MC**, **Fitzpatrick DA**, **de Groot PWJ**, **Harris D**, **Hoyer LL**, **Hube B**, **Klis FM**, **Kodira C**, **Lennard N**, **Logue ME**, **Martin R**, **Neiman AM**, **Nikolaou E**, **Quail MA**, **Quinn J**, **Santos MC**, **Schmitzberger FF**, **Sherlock G**, **Shah P**, **Silverstein KAT**, **Skrzypek MS**, **Soll D**, **Staggs R**, **Stansfield I**, **Stumpf MPH**, **Sudbery PE**, **Srikantha T**, **Zeng Q**, **Berman J**, **Berriman M**, **Heitman J**, **Gow NAR**, **Lorenz MC**, **Birren BW**, **Kellis M**, **Cuomo CA**. 2009. Evolution of pathogenicity and sexual reproduction in eight *Candida* genomes. Nature **459**:657–62.

3. **Noble SM**, **Johnson AD**. 2005. Strains and strategies for large-scale gene deletion studies of the diploid human fungal pathogen *Candida albicans*. Eukaryot. Cell **4**:298–309.

4. **Staib P**, **Morschhäuser J**. 1999. Chlamydospore formation on staib agar as a species-specific characteristic of *Candida dubliniensis*. Mycoses **42**:521–524.

5. **Cormack BP**, **Falkow S**. 1999. Efficient homologous and illegitimate recombination in the opportunistic yeast pathogen *Candida glabrata*. Genetics **151**:979–87.

6. **Giaever G**, **Chu AM**, **Ni L**, **Connelly C**, **Riles L**, **Véronneau S**, **Dow S**, **Lucau-Danila A**, **Anderson K**, **André B**, **Arkin AP**, **Astromoff A**, **El Bakkoury M**, **Bangham R**, **Benito R**, **Brachat S**, **Campanaro S**, **Curtiss M**, **Davis K**, **Deutschbauer A**, **Entian K-**

D, **Flaherty P**, **Foury F**, **Garfinkel DJ**, **Gerstein M**, **Gotte D**, **Güldener U**, **Hegemann JH**, **Hempel S**, **Herman Z**, **Jaramillo DF**, **Kelly DE**, **Kelly SL**, **Kötter P**, **LaBonte D**, **Lamb DC**, **Lan N**, **Liang H**, **Liao H**, **Liu L**, **Luo C**, **Lussier M**, **Mao R**, **Menard P**, **Ooi SL**, **Revuelta JL**, **Roberts CJ**, **Rose M**, **Ross-Macdonald P**, **Scherens B**, **Schimmack G**, **Shafer B**, **Shoemaker DD**, **Sookhai-Mahadeo S**, **Storms RK**, **Strathern JN**, **Valle G**, **Voet M**, **Volckaert G**, **Wang C**, **Ward TR**, **Wilhelmy J**, **Winzeler EA**, **Yang Y**, **Yen G**, **Youngman E**, **Yu K**, **Bussey H**, **Boeke JD**, **Snyder M**, **Philippsen P**, **Davis RW**, **Johnston M**. 2002. Functional profiling of the *Saccharomyces cerevisiae* genome. Nature **418**:387–391.

7. **Granger DL**, **Perfect JR**, **Durack DT**. 1985. Virulence of *Cryptococcus neoformans.* Regulation of capsule synthesis by carbon dioxide. J. Clin. Invest. **76**:508–16.

8. **Kim SH**, **Iyer KR**, **Pardeshi L**, **Muñoz JF**, **Robbins N**, **Cuomo CA**, **Wong KH**, **Cowen LE**. 2019. Genetic analysis of *Candida auris* implicates Hsp90 in morphogenesis and azole tolerance and Cdr1 in azole resistance. MBio **10**:e02529-18.

9. **Geng J**, **Nair U**, **Yasumura-Yorimitsu K**, **Klionsky DJ**. 2010. Post-Golgi Sec proteins are required for autophagy in *Saccharomyces cerevisiae*. Mol. Biol. Cell **21**:2257–69.

10. **Piotrowski JS**, **Li SC**, **Deshpande R**, **Simpkins SW**, **Nelson J**, **Yashiroda Y**, **Barber JM**, **Safizadeh H**, **Wilson E**, **Okada H**, **Gebre AA**, **Kubo K**, **Torres NP**, **LeBlanc MA**, **Andrusiak K**, **Okamoto R**, **Yoshimura M**, **DeRango-Adem E**, **van Leeuwen J**, **Shirahige K**, **Baryshnikova A**, **Brown GW**, **Hirano H**, **Costanzo M**, **Andrews B**, **Ohya Y**, **Osada H**, **Yoshida M**, **Myers CL**, **Boone C**. 2017. Functional annotation of chemical libraries across diverse biological processes. Nat. Chem. Biol. **13**:982–993.

11. **Veri AO**, **Miao Z**, **Shapiro RS**, **Tebbji F**, **O'Meara TR**, **Kim SH**, **Colazo J**, **Tan K**,

Vyas VK, **Whiteway M**, **Robbins N**, **Wong KH**, **Cowen LE**. 2018. Tuning Hsf1 levels drives distinct fungal morphogenetic programs with depletion impairing Hsp90 function and overexpression expanding the target space. PLoS Genet. **14**:e1007270.

12. **Norrander J**, **Kempe T**, **Messing J**. 1983. Construction of improved M13 vectors using oligodeoxynucleotide-directed mutagenesis. Gene **26**:101–106.
